# Evidence for a Widespread Third System for Bacterial Polysaccharide Export across the Outer Membrane Comprising a Composite OPX/β-Barrel Translocon

**DOI:** 10.1128/mbio.02032-22

**Published:** 2022-08-16

**Authors:** Johannes Schwabe, María Pérez-Burgos, Marco Herfurth, Timo Glatter, Lotte Søgaard-Andersen

**Affiliations:** a Department of Ecophysiology, Max Planck Institute for Terrestrial Microbiology, Marburg, Germany; b Core Facility for Mass Spectrometry & Proteomics, Max Planck Institute for Terrestrial Microbiology, Marburg, Germany; Institut Pasteur

**Keywords:** beta-barrel proteins, export of polysaccharides, *Myxococcus xanthus*, OM translocon, OPX proteins, synthase-dependent pathway, Wzx/Wzy pathway, capsular polysaccharide, exopolysaccharide

## Abstract

In Gram-negative bacteria, secreted polysaccharides have multiple critical functions. In Wzx/Wzy- and ABC transporter-dependent pathways, an outer membrane (OM) polysaccharide export (OPX) type translocon exports the polysaccharide across the OM. The paradigm OPX protein Wza of Escherichia coli is an octamer in which the eight C-terminal domains form an α-helical OM pore and the eight copies of the three N-terminal domains (D1 to D3) form a periplasmic cavity. In synthase-dependent pathways, the OM translocon is a 16- to 18-stranded β-barrel protein. In Myxococcus xanthus, the secreted polysaccharide EPS (exopolysaccharide) is synthesized in a Wzx/Wzy-dependent pathway. Here, using experiments, phylogenomics, and computational structural biology, we identify and characterize EpsX as an OM 18-stranded β-barrel protein important for EPS synthesis and identify AlgE, a β-barrel translocon of a synthase-dependent pathway, as its closest structural homolog. We also find that EpsY, the OPX protein of the EPS pathway, consists only of the periplasmic D1 and D2 domains and completely lacks the domain for spanning the OM (herein termed a ^D1D2^OPX protein). *In vivo*, EpsX and EpsY mutually stabilize each other and interact in *in vivo* pulldown experiments supporting their direct interaction. Based on these observations, we propose that EpsY and EpsX make up and represent a third type of translocon for polysaccharide export across the OM. Specifically, in this composite translocon, EpsX functions as the OM-spanning β-barrel translocon together with the periplasmic ^D1D2^OPX protein EpsY. Based on computational genomics, similar composite systems are widespread in Gram-negative bacteria.

## INTRODUCTION

Most bacteria secrete one or more polysaccharides. These polysaccharides protect against environmental stresses and phage infection, contribute to surface colonization and biofilm formation, have important functions in beneficial and pathogenic human-, animal- and plant-microbe interactions, provide the basis for serotyping and several antibacterial vaccines, and have many applications in the food, pharmaceutical, and medical industries (1–3). Here, we focus on the export of polysaccharides across the outer membrane (OM) in Gram-negative bacteria.

Secreted polysaccharides are large, chemically diverse molecules. Their synthesis hinges on three mechanisms, and their export across the OM in Gram-negative bacteria hinges on two known mechanisms (4, 5). In Wzx/Wzy-dependent pathways, biosynthesis is initiated on the cytoplasmic side of the inner membrane (IM) by a phosphoglycosyltransferase (PGT). Subsequently, glycosyltransferases (GTs) add monosaccharides to generate the repeat unit. The Wzx flippase “flips” individual repeat units across the IM to the periplasm, where the Wzy polymerase polymerizes them. On the periplasmic side, the polysaccharide copolymerase (PCP), an integral IM protein with an extended periplasmic domain, regulates polymerization and polysaccharide transfer across the periplasm to the OM (5, 6). A protein of the OM polysaccharide export (OPX) family exports the polysaccharide across the OM (7, 8). Wza of Escherichia coli is the best-studied OPX protein. In contrast to other pore-forming OM proteins, the octameric Wza spans the OM using an α-helical barrel (7). The periplasmic part of the OPX protein interacts with the oligomeric PCP to establish a conduit for the polysaccharide to reach the OM (6, 8–10). In ABC transporter-dependent pathways, synthesis of the polysaccharide is also initiated on the cytoplasmic side of the IM and fully completed before translocation across the IM by an ABC transporter (5). Transfer across the periplasm involves a PCP, and the polysaccharide is exported across the OM by an OPX protein (5, 6). Synthase-dependent pathways differ considerably from these two pathways and consist of only three core components (4). An IM-embedded synthase with GT activity synthesizes the polysaccharide and, in parallel, facilitates its translocation across the IM. A tetratricopeptide repeat (TPR)-containing protein contributes to polysaccharide transfer across the periplasm. The polysaccharide is exported across the OM through an integral OM 16- to 18-stranded β-barrel protein as described in structural work on PgaA and BcsC of E. coli and AlgE of Pseudomonas aeruginosa (11–13).

Here, we combine experiments, computational structural biology, and genomics to provide evidence for a novel type of polysaccharide OM export mechanism widespread in Gram-negative bacteria. In this system, a short OPX protein comprises only periplasmic domains and lacks the domain for spanning the OM functions together with an OM β-barrel protein to generate a composite OPX/β-barrel translocon.

## RESULTS AND DISCUSSION

### Myxobacterial gene clusters for secreted polysaccharides encode an OM β-barrel protein.

The Gram-negative deltaproteobacterium Myxococcus xanthus secretes three polysaccharides, i.e., exopolysaccharide (EPS), spore coat polysaccharide (SPS), and biosurfactant polysaccharide (BPS), using three dedicated Wzx/Wzy-dependent pathways (see Fig. S1A in the supplemental material) (14). In all three systems, the gene annotated as encoding the OPX protein (EpsY, ExoA, WzaB) is syntenic with a gene encoding a protein of unknown function (EpsX, ExoB, WzpB). This synteny is largely conserved in the orthologous myxobacterial gene clusters (Fig. S1A).

10.1128/mbio.02032-22.1 FIG S1 The gene clusters for EPS, SPS, and BPS biosynthesis in M. xanthus are conserved in other myxobacteria. (A) Left, 16S rRNA-based tree of fully sequenced myxobacteria. Right, a reciprocal best BLASTP hit method was used to identify orthologs as described previously (17, 45). Ten genes was considered the maximum distance for a gene to be in a cluster. For each pathway, genes in the same cluster are marked in the same color. Genes within a distance of <10 genes were considered part of the same cluster. Clusters were considered separate when they were more than 10 genes apart. Conserved but orphan genes are colored in light gray. Genes without an ortholog are indicated by an X. In the top row, M. xanthus genes are color coded according to the code shown below the chart. Black arrows indicate synteny of genes encoding the OPX protein (purple) and genes encoding an 18-stranded β-barrel protein (teal) conserved in *eps*, *sps*, and *bps* gene clusters in myxobacteria. In the *eps* gene cluster, WzeX is important for EPS synthesis and was proposed to act as the BY kinase partner of EpsV (14, 18), the serine *O*-acetyltransferase EpsC is thought to be involved in sugar nucleotide precursor biosynthesis and is not important for EPS biosynthesis (17–19), and EpsB is a predicted glycoside hydrolase, which is not important for EPS synthesis (19). EpsW is a response regulator involved in regulation of EPS synthesis (70). *epsG* and *epsF* are annotated as encoding a magnesium transporter and a hybrid response regulator/histidine kinase, respectively, and *epsF* is not required for EPS synthesis (19). In the *bps* cluster, *wzcB* encodes a PCP protein with an N-terminal BY kinase domain. ExoA/ExoB are sometimes referred to as WzaS/WzpS, and EpsY/EpsX are sometimes referred to as WzaX/WzpX (18, 20). For a more detailed description of proteins encoded by the *sps* and *bps* gene clusters and not described in the color code, see references 17, 18, and 45. (B) Operon structure of the two gene clusters for *eps* synthesis. The lower diagram shows genes in the same color code used in panel A. Kinked arrows indicate transcription start sites as mapped in reference 48. The upper diagram shows data from RNA-seq as base-by-base alignment coverage for the two *eps* loci on the total RNA isolated from cells growing in 1% CTT (48). Positive and negative values indicate reads mapped to the forward and reverse strands, respectively. Reads assigned to a gene are colored according to the gene color code; intergenic regions are in gray. Numbers in genes represent MXAN_ locus tags. Gene names are shown at the bottom of the panel. Download FIG S1, PDF file, 0.5 MB. Copyright © 2022 Schwabe et al. 2022 Schwabe et al. https://creativecommons.org/licenses/by/4.0/ This content is distributed under the terms of the Creative Commons Attribution 4.0 International license.

**FIG 1:**
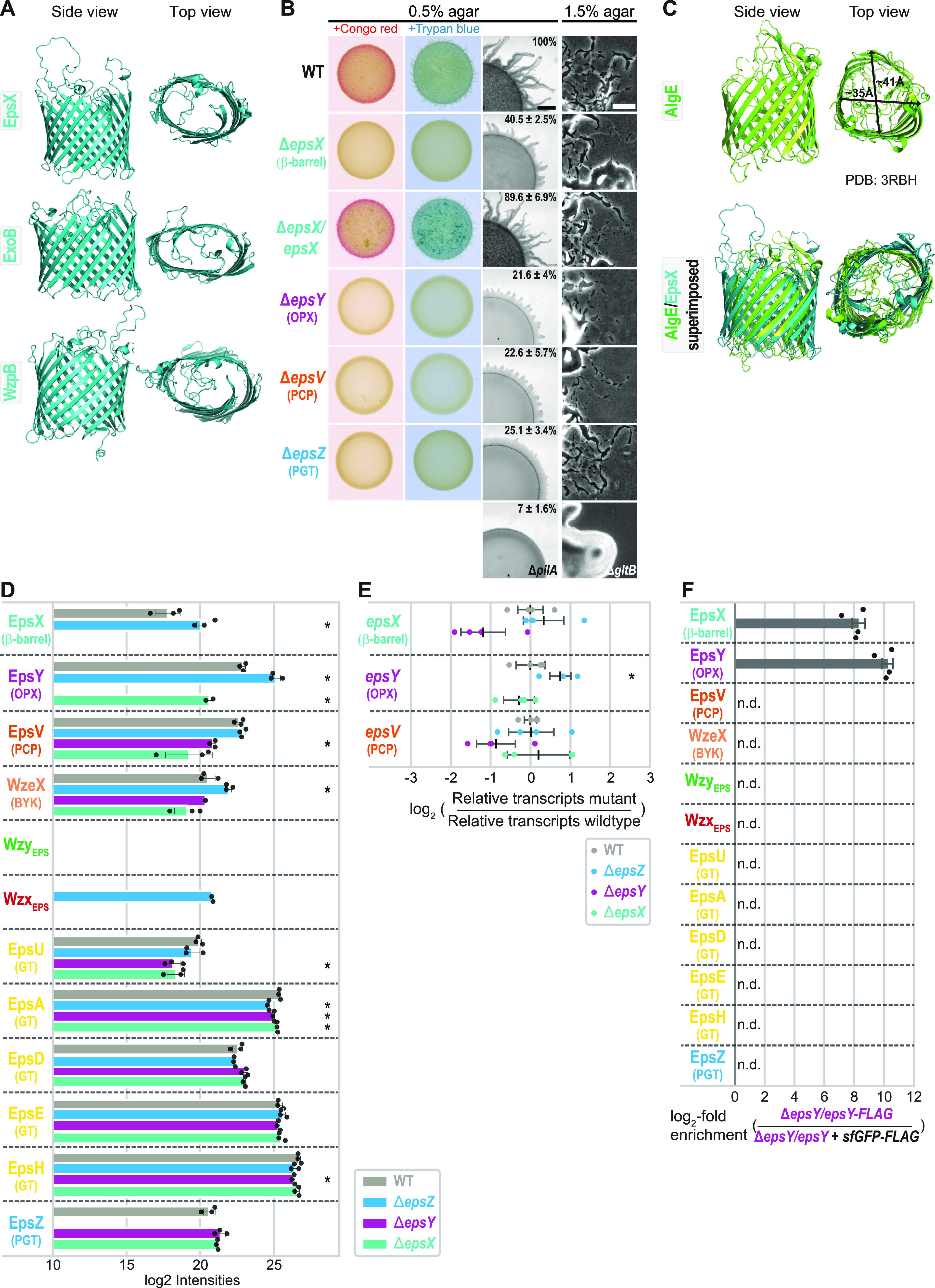
The 18-stranded β-barrel protein EpsX is an integral part of the EPS pathway. (A) AlphaFold models of EpsX, ExoB, and WzpB. Proteins are oriented based on the N and C termini of OM β-barrel proteins being periplasmic (66). Model ranks 1 are shown. (B) Phenotypic characterization of Δ*epsX* mutant. Two left columns, cells were placed on 0.5% agar supplemented with 0.5% CTT and Congo red or trypan blue and incubated for 24 h. Two right columns, T4P-dependent motility and gliding motility were tested on 0.5% and 1.5% agar, respectively, supplemented with 0.5% CTT, and images were recorded after 24 h. The Δ*pilA* mutant, which lacks the major pilin of the T4P (67), and the Δ*gltB* mutant, which lacks a component of the gliding motility machinery (23), served as negative controls for T4P-dependent and gliding motility, respectively. In the complementation strain, *epsX* was expressed from the *pilA* promoter on a plasmid integrated in a single copy at the Mx8 *attB* site. Scale bars, 1 mm (left) and 50 μm (right). Numbers indicate expansion from the edge of the colony calculated from three biological replicates and relative to that of the WT, where 100% corresponds to 1.4 mm. (C) Comparison of AlgE and EpsX. Upper panel, lateral and top views of the solved structure of AlgE (PDB 3RBH) (11). Arrows indicate the external diameter of the β-barrel. Lower panel, superimposition of the solved structure of AlgE and the EpsX AlphaFold model. EpsX is colored in teal. EpsX aligns to AlgE with a root mean square deviation (RMSD) of 6.035 Å over 1,501 C*_α_*. (D) EpsX and EpsY mutually stabilize each other, and EpsY stabilizes EpsV. Protein amounts in whole-cell proteomes of M. xanthus strains were determined using LFQ mass spectrometry-based proteomics (see Materials and Methods). Normalized log_2_ intensities of Eps proteins in the indicated strains are shown. Missing bars indicate that the proteins were not detected. Data points represent three biological replicates. Error bars, standard deviation (SD) based in these replicates. *, *P < *0.05 (Welch’s test). WzeX is important for EPS synthesis and was proposed to act as the BY kinase partner of EpsV (14, 18). (E) RT-qPCR analysis of *epsV*, *epsY*, and *epsX* transcripts levels. Total RNA was isolated from cells grown as panel D. Data are shown as log_2_ transcripts in a mutant relative to that of the WT. Individual data points represent four biological replicates with each two technical replicates and are colored according to the strain analyzed. Center marker and error bars represent mean and SD. *, *P < *0.05 (Welch’s test). (F) EpsX and EpsY interact. Pulldown experiments on whole-cell lysates of strains expressing EpsY-FLAG or sfGFP-FLAG (negative control). In the EpsY-FLAG strain, *epsY-FLAG* was expressed ectopically from the *pilA* promoter on a plasmid integrated in a single copy at the Mx8 *attB* site. In the sfGFP-FLAG strain, *epsY* was expressed from the *pilA* promoter on a plasmid integrated in a single copy at the Mx8 *attB* site, and *sfGFP-FLAG* was expressed from the *pilA* promoter on a plasmid integrated in a single copy at the *18-19* intergenic locus. Samples from four biological replicates were analyzed by LC-MS (see Material and Methods). Log_2_-fold enrichment of proteins in EpsY-FLAG over sfGFP-FLAG samples was calculated. Columns represent mean log_2_-fold enrichment (*n *= 4). Error bars, SD based on these replicates; n.d., proteins detected in neither EpsY-FLAG nor sfGFP-FLAG samples.

EpsX, ExoB, and WzpB have a type 1 signal peptide based on sequence analysis. AlphaFold structural models (see Materials and Methods) predict with high accuracy that they fold into 18-stranded antiparallel β-barrels with an elliptical shape and a central channel (Fig. 1A; Fig. S2A to C). As expected, the parts of the proteins predicted with low confidence correspond to extracellular loops connecting the antiparallel β-strands (Fig. S2A to C). The three AlphaFold models could readily be superimposed (Fig. S2D), documenting that the proteins have the same fold overall. ExoB is important for SPS biosynthesis by an unknown mechanism (15), and WzpB is an OM protein (16), but it is not known whether it is important for BPS synthesis. Together, these data support that EpsX, ExoB, and WzpB are integral OM 18-stranded β-barrel proteins. To investigate the function of these proteins and their orthologs, we focused on EpsX.

10.1128/mbio.02032-22.2 FIG S2 AlphaFold models of EpsX, ExoB, and WzpB. (A to C) pLDDT (predicted local distance difference test) and pAE (predicted alignment error) plots for five models of EpsX (A), ExoB (B), and WzpB (C) as predicted by AlphaFold. For all three proteins, model rank 1 (marked by a green box) was used for further analysis and is shown colored based on the pLDDT values. Mobility and low sequence conservation are typical features of the extracellular loops of β-barrels (66), likely explaining the low spatial confidence in loop positioning in the EpsX, ExoB, and WzpB models. Signal peptides were removed before generating a model. (D) Superimposition of AlphaFold-predicted structures of EpsX and ExoB and of EpsX and WzpB. Left panel, EpsX is colored in gray and ExoB in teal. ExoB aligns to EpsX with an RMSD of 3.427Å over 232 C*_α_*. Right panel, EpsX is colored in gray and MXAN_1916 in teal. WzpB aligns to EpsX with an RMSD of 4.484Å over 320 C*_α_*. (E) Solved structure of Wzi (PDB 2YNK) (22). The protein is colored in light pink, except for the N-terminal α-helical bundle that closes the barrel to the periplasm (22), which is colored in light orange. Download FIG S2, PDF file, 2.4 MB. Copyright © 2022 Schwabe et al. 2022 Schwabe et al. https://creativecommons.org/licenses/by/4.0/ This content is distributed under the terms of the Creative Commons Attribution 4.0 International license.

### EpsX is important for EPS biosynthesis.

To investigate the function of EpsX, we generated an in-frame deletion in *epsX* (Δ*epsX*). Using plate-based colorimetric assays with Congo red or trypan blue as readouts of EPS synthesis, we observed that the wild type (WT) synthesized EPS. The Δ*epsX* mutation, similarly to the Δ*epsZ*, Δ*epsV*, and Δ*epsY* mutations that inactivate genes for proteins in the EPS pathway (Fig. S1A and B) (17–19), caused strongly reduced EPS synthesis (Fig. 1B). EPS is important for type IV pilus (T4P)-dependent motility (14). Consistently, while the WT formed colonies with long flares at the edge characteristic of T4P-dependent motility and the Δ*pilA* negative control formed smooth-edged colonies, the Δ*epsX* mutant generated colonies with short flares, although these were slightly longer than those previously observed for the Δ*epsZ*, Δ*epsV*, and Δ*epsY* mutants (17–19) (Fig. 1B). The Δ*epsX* mutant was recently described as having a minor defect in T4P-dependent motility and, in agreement with our observations, to be less impaired in T4P-dependent motility than a Δ*epsY* mutant (20); we attribute the differences to different strain backgrounds and conditions of the motility assay. The Δ*epsX* mutant, similarly to the WT and the Δ*epsZ*, Δ*epsV*, and Δ*epsY* mutants, displayed the single cells at the colony edge characteristic of gliding motility, while the Δ*gltB* negative control did not (Fig. 1B). To test for polar effects of the Δ*epsX* mutation (Fig. S1B), we performed complementation experiments in which *epsX* was expressed ectopically in the Δ*epsX* mutant. Ectopic expression of *epsX*, as previously shown for *epsZ*, *epsV*, and *epsY* in the respective mutants (17), complemented the defects in EPS biosynthesis and T4P-dependent motility in the Δ*epsX* mutant (Fig. 1B). We conclude that EpsX is important for EPS synthesis and likely an integral component of the EPS pathway.

In the E. coli and Klebsiella pneumoniae Wzx/Wzy-dependent pathways for capsule biosynthesis, the OM 18-stranded β-barrel protein Wzi is important for cell surface anchoring of the capsule but not for its biosynthesis or its export (21, 22), arguing that Wzi and EpsX have different functions. Wzi and EpsX share 16.8% sequence identity and 22.5% sequence similarity. Moreover, while the β-barrel in the solved structure of Wzi is circular (22) (Fig. S2E), the EpsX β-barrel in the AlphaFold model is elliptical (Fig. 1A). Wzi also contains an N-terminal α-helical bundle that occludes the periplasmic side of the β-barrel and extracellular loops that fold into and occlude the β-barrel on the extracellular side (22). In contrast, EpsX lacks the N-terminal α-helical bundle, and the extracellular loops, although modeled with low confidence (Fig. S2A), do not fold into the β-barrel, indicating that EpsX is open to the periplasm and could also, when required, open to the cell exterior. This structural comparison also supports the conclusion that EpsX has a function different from that of Wzi.

Subsequently, by searching for structural homologs of EpsX using Foldseek (see Materials and Methods), we identified the OM translocon AlgE of the synthase-dependent pathway for alginate export in P. aeruginosa (11) as the closest structural homolog. The two proteins share 13.4% sequence identity and 20.1% sequence similarity. Similar to EpsX in the AlphaFold model, the 18-stranded β-barrel of AlgE in the solved structure has an elliptical shape (Fig. 1C). The two proteins could readily be superimposed except for extracellular loops in AlgE that fold into the β-barrel and partially occlude the pore (Fig. 1C).

### EpsX and EpsY mutually stabilize each other and interact.

There are several reported examples in M. xanthus of directly interacting OM, periplasmic, and IM proteins that stabilize each other (23–26). Consequently, to identify potential EpsX interaction partners, we performed whole-cell label-free quantitative (LFQ) mass spectrometry-based proteomics (see Materials and Methods) focusing on the Eps proteins.

In the WT, we detected all Eps proteins except for the integral IM proteins Wzx_EPS_ and Wzy_EPS_ (Fig. 1D). In the Δ*epsX* mutant, the OPX protein EpsY accumulated at a significantly reduced level (Fig. 1D). Conversely, in the Δ*epsY* mutant, EpsX was not detected, and accumulation of the PCP EpsV was significantly reduced (Fig. 1D). In contrast, the Δ*epsZ* mutant, which lacks the PGT for initiating EPS synthesis, had slightly decreased (EpsA), increased (Wzx_EPS_, EpsY, and EpsX), or WT levels of the Eps proteins (Fig. 1D). These observations support that the decreased accumulation of EpsY in the Δ*epsX* mutant and of EpsX and EpsV in the Δ*epsY* mutant is not caused by lack of EPS biosynthesis and export *per se*. Some GTs are also slightly but significantly reduced in the Δ*epsX* and Δ*epsY* mutants (Fig. 1D). Here, we focused on the significant accumulation dependency of EpsX, EpsY, and EpsV. Importantly, and in agreement with the previously reported complementation experiments of the Δ*epsX* and Δ*epsY* mutants (Fig. 1B) (17), reverse transcriptase quantitative PCR (RT-qPCR) provided evidence that the changes in EpsX, EpsY, and EpsV levels are independent of transcription in the Δ*epsX* and Δ*epsY* mutants (Fig. 1E). We conclude that the OM β-barrel protein EpsX and the OPX protein EpsY mutually stabilize each other and that EpsY stabilizes the PCP EpsV in the IM. These findings agree with OPX and PCP proteins interacting in the periplasm (8–10). They also support the idea that EpsX, EpsY, and EpsV form a complex that spans the cell envelope.

To address whether EpsX and EpsY interact, we performed pulldown mass spectrometry experiments (see Materials and Methods) using an Δ*epsY* strain ectopically expressing an EpsY protein with a FLAG tag inserted at residue 43 between two periplasmic domains (herein termed EpsY-FLAG) (see details below). As a negative control, we used the Δ*epsY* strain ectopically expressing EpsY without a FLAG tag and a superfolder green fluorescent protein (sfGFP)-FLAG protein. Both FLAG-tagged proteins accumulated, and EpsY-FLAG supported EPS synthesis and T4P-dependent motility, demonstrating that the FLAG tag did not disrupt EpsY’s functional interactions with other proteins (Fig. S3A and B). Importantly, among proteins involved in EPS synthesis, only EpsX was highly enriched in the EpsY-FLAG pulldown experiments (Fig. 1F; Fig. S3C and D).

10.1128/mbio.02032-22.3 FIG S3 EpsX and EpsY interact in *in vivo* pulldown experiments. (A) Immunoblot detection of FLAG-tagged proteins from strains of the indicated genotypes. Total cell extract from the same number of cells was loaded per lane, except for Δ*epsY*/*epsY*+*sfGFP-FLAG*, which was diluted 1:3 in SDS lysis buffer. In the complementation strains, *epsY* and *epsY-FLAG* were expressed ectopically from the *pilA* promoter on plasmids integrated in a single copy at the Mx8 *attB* site. In the Δ*epsY*/*epsY*+*sfGFP-FLAG* strain, *sfGFP-FLAG* was expressed from the *pilA* promoter on a plasmid integrated in a single copy at the *18-19* intergenic locus. The top blot was probed with anti-FLAG antibody, stripped, and probed with anti-PilC antibody as a loading control. (B) Phenotypic characterization of the Δ*epsY* mutant. Strains are the same as in panel A. To test for EPS synthesis, cells were placed on 0.5% agar supplemented with 0.5% CTT and Congo red or trypan blue as indicated and incubated for 24 h. To test for T4P-dependent motility, cells were placed on 0.5% agar supplemented with 0.5% CTT and images were recorded after 24 h. The Δ*epsZ* and Δ*pilA* mutants were used as negative controls for EPS synthesis and T4P-dependent motility, respectively. Percentages indicate expansion from the edge of the colony, calculated from three technical replicates and relative to that of the WT, where 100% corresponds to 0.9 mm. Scale bar, 1 mm. (C) Volcano plot of pulldown experiments with EpsY-FLAG and sfGFP-FLAG. For both strains (Δ*epsY*/*epsY*-*FLAG* and Δ*epsY*/*epsY*+*sfGFP-FLAG*), samples were prepared from four biological replicates and analyzed by LC-MS, and log_2_-fold ratios of EpsY-FLAG over the negative control sfGFP-FLAG were calculated (see Material and Methods). *x* axis, log_2_-fold ratios of proteins in EpsY-FLAG samples over sfGFP-FLAG samples. *y* axis, −log_10_ *P* value. Significantly enriched proteins in EpsY-FLAG (log_2_ enrichment ≥ 3; *P* value ≤ 0.001; −log_10_ *P* value ≥ 3) are indicated by red data points and listed in the table in panel D. Significance thresholds are indicated with dashed lines. Data points represent the means of four biological replicates. (D) Proteins significantly enriched in EpsY-FLAG samples compared to sfGFP-FLAG samples. Protein name, annotation, and type of signal peptide present are indicated. Download FIG S3, PDF file, 1.4 MB. Copyright © 2022 Schwabe et al. 2022 Schwabe et al. https://creativecommons.org/licenses/by/4.0/ This content is distributed under the terms of the Creative Commons Attribution 4.0 International license.

Together, the cooccurrence of EpsX and EpsY, the mutually stabilizing effect of EpsX and EpsY, and the pulldown experiments strongly support that EpsX and EpsY directly interact. The observations that EpsX and EpsY accumulate in the absence of EpsZ (Fig. 1D) and thus stabilize each other in the absence of EPS synthesis suggest that EpsX and EpsY interact independently of EPS synthesis and that this interaction is not transient. We note that the PCP EpsV was not enriched in the EpsY-FLAG pulldown experiments (Fig. 1F), while EpsV still accumulated in the absence of EpsZ (Fig. 1D). We speculate that EpsV was not detected in the pulldown experiments because it might not be efficiently extracted from the IM.

To investigate how EpsX, EpsY, and EpsV interact, we first analyzed EpsY and its homologs.

### Myxobacterial OPX proteins are short and comprise only two periplasmic domains.

**FIG 2:**
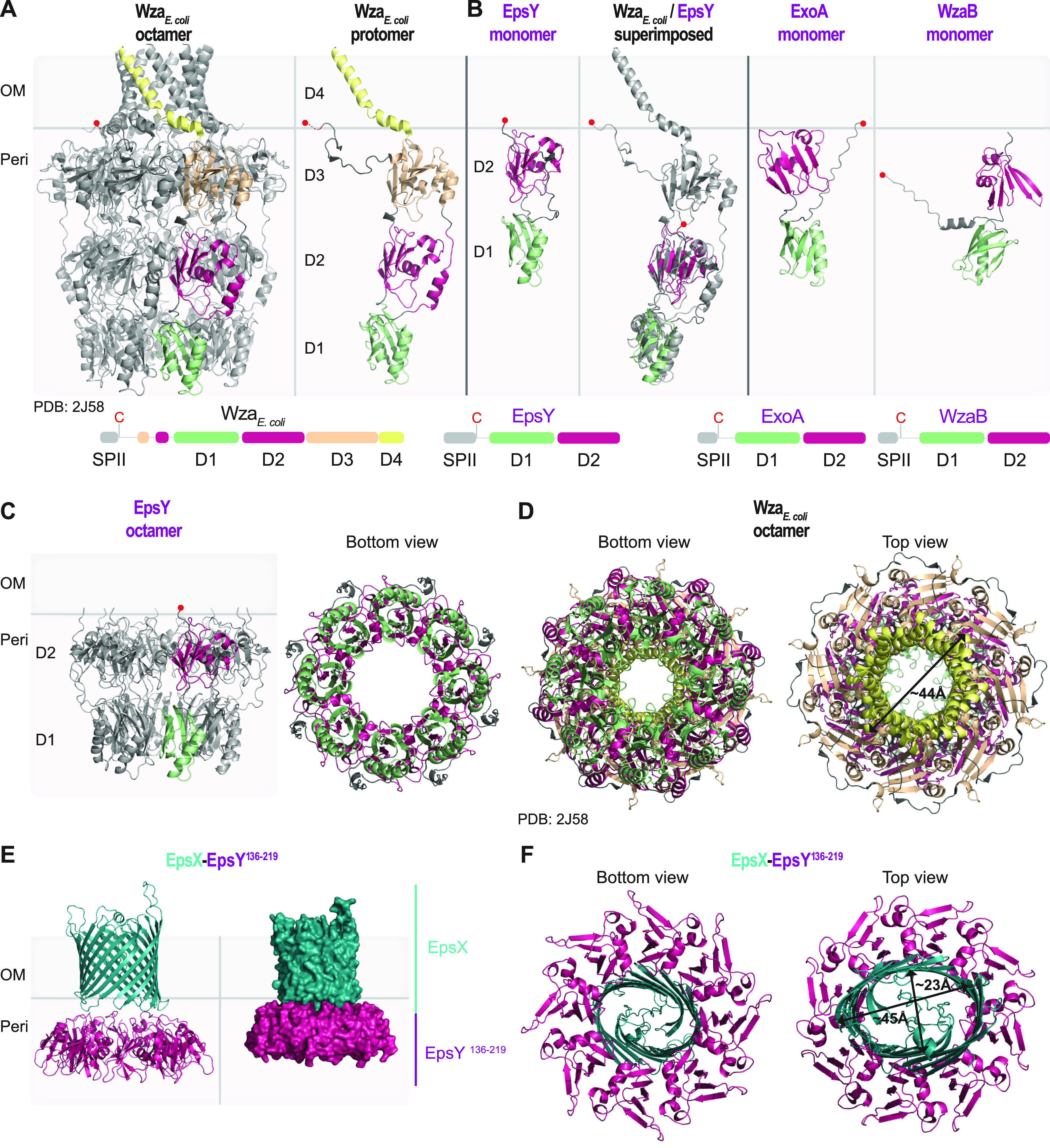
Structural characterization of the EpsY ^D1D2^OPX protein alone and in complex with EpsX, its partner 18-stranded β-barrel protein. (A) Structure of Wza_E. coli_. Left panel, the solved structure of octameric Wza (PDB 2J58) (7). Right panel, an individual Wza protomer. The four domains of Wza are labeled D1 to D4. Light green, D1; dark pink, D2; light orange, D3; yellow, D4. The acylated N-terminal cysteine is indicated by a red circle and placed at the inner leaflet of the OM. Lower panel, domain organization of Wza. (B) AlphaFold model of EpsY. Left panel, lateral view of EpsY monomer as predicted by AlphaFold. Right panel, superimposition of a Wza protomer from the solved structure (gray) and the EpsY model. The EpsY monomer aligns to the Wza protomer with an RMSD of 3.306 Å over 904 C*_α_*. Right panels, AlphaFold models of ExoA and WzaB monomers. In all three AlphaFold models, the two domains are labeled D1 and D2 and colored according to the homologous domains in Wza. The acylated N-terminal cysteine is indicated by a red circle; note that the acylated N-terminal cysteine of WzaB is not modeled “on top” of D2, but the confidence in the relative position of this residue is low (Fig. S3C). Model rank 1 is shown for all structures. Lower panels, domain organization of EpsY, ExoA, and WzaB. SPII, type 2 signal peptide. (C) AlphaFold-Multimer model of octameric EpsY. Left panel, one protomer is colored as described in the legend for panel B. Right panel, bottom view of octameric EpsY with all protomers colored as described for panel B. Model rank 1 is shown. (D) Structure of Wza. All eight protomers are colored as described in the legend for panel A. An arrow indicates the external diameter of the α-helical pore. In the bottom view, the tyrosine residues that form the so-called tyrosine ring are present in the loops extending into the central channel. (E and F) AlphaFold-Multimer model of a heterocomplex of octameric EpsY^136-219^ and an EpsX monomer. In the heterocomplex, the EpsY^136-219^ octamer is colored as D2 as described for panel B, and EpsX is colored teal. (E) The right panel is a surface-rendered representation. Model rank 1 is shown. (F) Arrows indicate the diameter of the β-barrel. Model rank 1 is shown.

In the Wza_E. coli_ octamer, individual protomers have four structural domains (D1 to D4) (7) (Fig. 2A). The N-terminal domains D1 to D3 are periplasmic, with D1 containing the characteristic polysaccharide export sequence (PES) motif. D2 and D3 are structurally related and have a β-grasp fold (27, 28). The C-terminal D4 is an amphipathic α-helix, which is inserted in the OM. In the octamer, the eight copies of D1 to D3 generate a periplasmic cavity, and the eight C-terminal α-helices create the α-helical barrel in the OM (Fig. 2A). Wza has a type 2 signal peptide and is N-terminally acylated (7), which is important for OM integration (27, 29).

Based on sequence analysis and a high-accuracy AlphaFold structural model, EpsY contains only D1 with the PES motif and D2 while lacking D3 and, most strikingly, D4 (Fig. 2B; Fig. S4A). The two EpsY domains could readily be superimposed on the corresponding domains in a Wza protomer (Fig. 2B). Using AlphaFold-Multimer (see Materials and Methods), EpsY with high accuracy could generate an octamer in which D1 and D2 form a stacked, ring-like structure with a central cavity, similar to the Wza counterparts (Fig. 2C; compare Fig. 2A and D; Fig. S5A). The Wza octamer is closed at the periplasmic base of D1 by a so-called tyrosine ring (Fig. 2D); however, this tyrosine ring is lacking in the EpsY octamer (Fig. 2C). As in the monomer model (Fig. 2B), the acylated N-terminal cysteine would be placed on top of D2 (Fig. 2C), facilitating the OM association of octameric EpsY. From here on, we refer to OPX proteins that comprise only D1 and D2 as ^D1D2^OPX proteins.

10.1128/mbio.02032-22.4 FIG S4 AlphaFold models of EpsY, ExoA, and WzaB. (A to C) pLDDT and pAE plots for five models of the indicated proteins as predicted by AlphaFold. For all three proteins, model rank 1 (marked by a green box) was used for further analysis and is shown colored based on the pLDDT values. Signal peptides were removed before generating a model. Download FIG S4, PDF file, 1.6 MB. Copyright © 2022 Schwabe et al. 2022 Schwabe et al. https://creativecommons.org/licenses/by/4.0/ This content is distributed under the terms of the Creative Commons Attribution 4.0 International license.

10.1128/mbio.02032-22.5 FIG S5 AlphaFold-Multimer models of EpsY octamer, EpsX/EpsY^136-219^, and AlgE/EpsY^136-219^. (A, B, and D) pLDDT and pAE plots for five models of the indicated protein complexes as predicted by AlphaFold-Multimer. For all three complexes, model rank 1 (marked by a green box) was used for further analysis and is shown colored based on the pLDDT values. Signal peptides were removed before generating a model. (C) Surface charge of EpsX and octameric EpsY^136-219^ (contoured from +5 to −5 kT e^−1^). Negative and positive charges are colored red and blue, respectively. Electrostatics were calculated using the APBS plugin in PyMOL (see Material and Methods). Download FIG S5, PDF file, 2.8 MB. Copyright © 2022 Schwabe et al. 2022 Schwabe et al. https://creativecommons.org/licenses/by/4.0/ This content is distributed under the terms of the Creative Commons Attribution 4.0 International license.

Remarkably, EpsY orthologs in other myxobacteria are also of the ^D1D2^OPX type (Fig. S6). In addition, the OPX proteins for SPS (ExoA) and BPS (WzaB) export, as well as their orthologs, are of the ^D1D2^OPX type (Fig. 2B; Fig. S4B and C and S6). Except for one, all these ^D1D2^OPX proteins have a type 2 signal peptide and are predicted lipoproteins (Fig. S6). Thus, all 48 identified OPX proteins in myxobacteria are ^D1D2^OPX proteins and, most strikingly, lack D4 for generating the OM α-helical pore (Fig. S6). Consistent with these findings, a previous classification of OPX proteins (6) defined a subgroup consisting of short OPX proteins that included EpsY and ExoA.

10.1128/mbio.02032-22.6 FIG S6 Conservation of domain structure of ^D1D2^OPX proteins in myxobacteria. Excerpt from Fig. S1A focusing on the synteny of ^D1D2^OPX protein-encoding genes (purple) and OM 18-stranded β-barrel protein-encoding genes (teal) in the *eps*, *sps*, and *bps* gene clusters in myxobacterial genomes. In the right panel of each pathway, the domain architecture of the ^D1D2^OPX proteins is colored as described in the legend for Fig. 2A and B. Download FIG S6, PDF file, 0.2 MB. Copyright © 2022 Schwabe et al. 2022 Schwabe et al. https://creativecommons.org/licenses/by/4.0/ This content is distributed under the terms of the Creative Commons Attribution 4.0 International license.

### A ^D1D2^OPX protein and an 18-stranded β-barrel protein may form a translocon for OM polysaccharide export.

^D1D2^OPX proteins lack the domain for spanning the OM and, generally, cooccur with 18-stranded β-barrel proteins (Fig. S1A and S6). Moreover, the 18-stranded β-barrel proteins are structurally similar to the OM translocon AlgE (Fig. 1C), indicating that they can support polysaccharide export across the OM. The ^D1D2^OPX protein EpsY and the 18-stranded β-barrel protein EpsX mutually stabilize each other and interact in *in vivo* pulldown experiments, supporting that they interact directly. Based on these four lines of evidence, we hypothesized that a ^D1D2^OPX protein functions together with a β-barrel protein to create a composite ^D1D2^OPX/β-barrel translocon.

To test this hypothesis, we intended to generate a heterocomplex consisting of eight EpsY molecules and one EpsX molecule using AlphaFold-Multimer. However, this amount of sequence information is computationally highly demanding to analyze. Instead, we generated a heterocomplex consisting of EpsY D2 (amino acids [aa] 136 to 219), which is predicted to be close to the OM (Fig. 2B and C), and full-length EpsX. In a high-accuracy AlphaFold-Multimer structural model, EpsX is placed on top of D2 in the EpsY^136-219^ octamer (Fig. 2E; Fig. S5B) in an arrangement strikingly similar to that of the α-helical barrel “on top” of D3 in the Wza octamer (Fig. 2A). Also, the periplasmic region of EpsX and the OM-facing regions of D2 in the EpsY^136-219^ octamer have opposite surface charges (Fig. S5C). Moreover, the diameters of the α-helical barrel in Wza and the EpsX β-barrel in the EpsX/EpsY^136-219^ heterocomplex are similar (Fig. 2D and F). To test the specificity of the AlphaFold-Multimer prediction of the EpsX/EpsY^136-219^ complex, we attempted to generate a model of an AlgE/EpsY^136-219^ heterocomplex. Even in the best of the five predicted models in which the individual AlgE and EpsY^136-219^ proteins are calculated to high accuracy based on predicted local distance difference test (pLDDT) values (see Material and Methods), the AlgE β-barrel did not associate with the octameric ring structure of EpsY^136-219^, and the accuracy in the relative positioning of AlgE to octameric EpsY^136-219^ is low, as evidenced by the high predicted alignment error (pAE) values (Fig. S5D). Thus, despite EpsX and AlgE being close structural homologs, the low accuracy of the AlgE/EpsY^136-219^ model suggests high specificity in the EpsX/EpsY^136-219^ heterocomplex, as expected for a functionally relevant protein complex.

Together, this computational approach, the mutually dependent stability of EpsX/EpsY, and the observation that EpsX and EpsY interact in *in vivo* pulldown experiments support that these two proteins form a complex. In this complex, EpsX spans the OM and EpsY is periplasmic and associated with the OM via the N-terminal lipid group and the interaction with EpsX.

### PCPs in M. xanthus may have an extended periplasmic domain.

**FIG 3:**
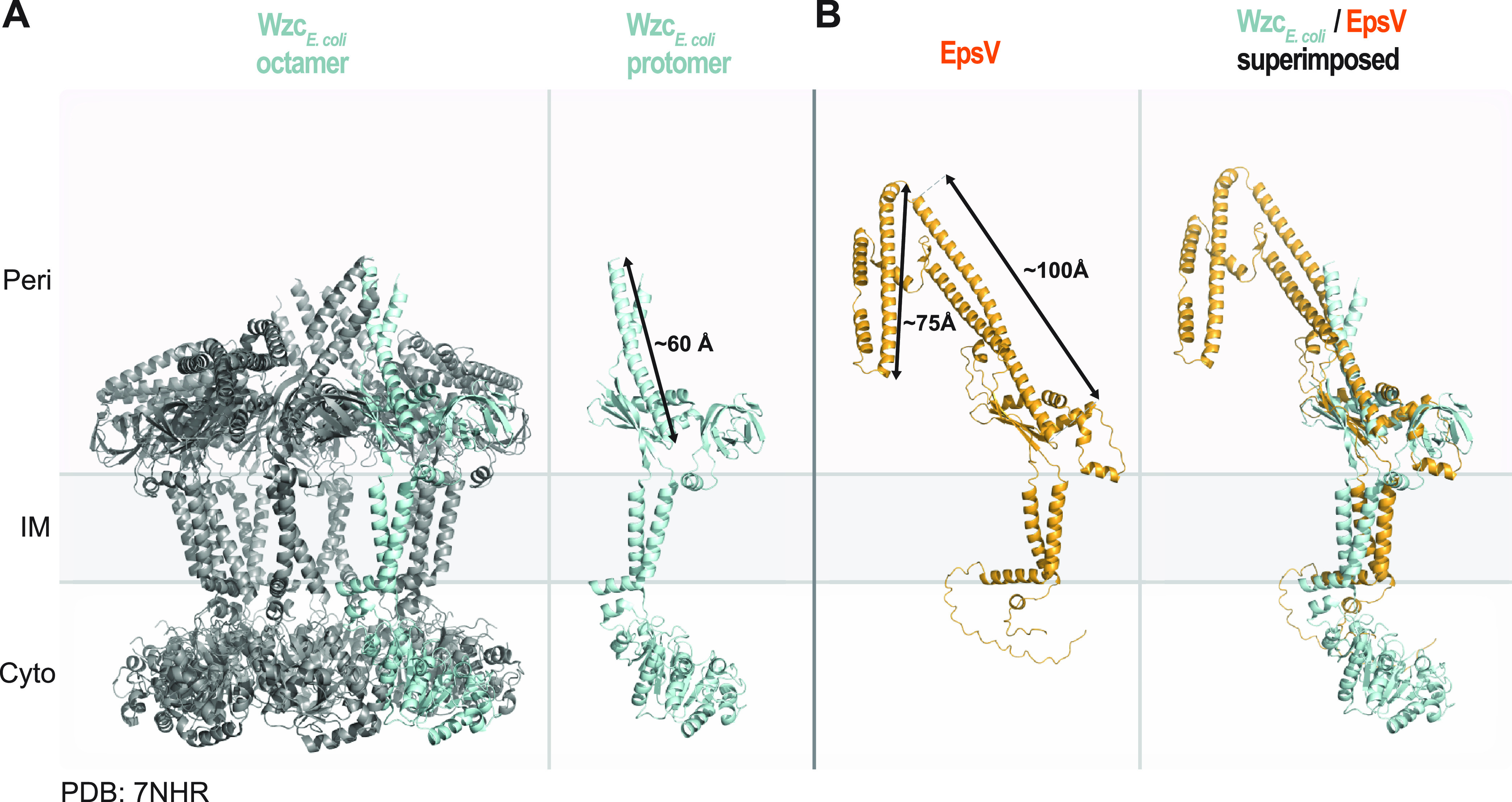
Structural characterization of the PCP EpsV. (A) Solved structure of octameric Wzc_E. coli_ (PDB 7NHR) (30). Left panel, the protein is colored in gray, and one protomer is colored in light blue. Right panel, individual Wzc protomer in class 2 conformation (30). Note that individual protomers have different conformations in the octamer. An arrow indicates the length of the extended α-helical stretch. (B) AlphaFold model of EpsV. Arrows indicate the lengths of the α-helical stretches. Model rank 1 is shown. Right panel, superimposition of a protomer from the solved structure of Wzc and the EpsV model. EpsV aligns to Wzc with an RMSD of 4.610 Å over 727 C*_α_*.

The IM PCP proteins interact with their cognate OPX proteins in the periplasm by using a region rich in α-helices (8–10, 30). In the case of the Wzc-Wza_E. coli_ complex, the PCP Wzc_E. coli_ in the solved structure extends ~60 Å from the IM into the periplasm (30) (Fig. 3A), while the periplasmic part of Wza extends ~100 Å from the OM into the periplasm, with each ring contributing ~30 Å (7). This gives an estimated periplasmic height of a Wzc-Wza complex of ~160 Å.

EpsY is important for the stability of the PCP EpsV (Fig. 1D), supporting that the two proteins interact. This raises the question of how the EpsY ^D1D2^OPX protein would be able to bridge the periplasm together with its EpsV PCP partner. A high-accuracy AlphaFold model of monomeric EpsV supports that it has two IM transmembrane α-helices and a periplasmic domain rich in α-helices (Fig. 3B; Fig. S7A), similar to other PCPs (6). The periplasmic part of EpsV is composed of two α-helical stretches with lengths of ~100 Å and ~75 Å, respectively, and connected by linkers. Depending on the conformation of the α-helical stretches in EpsV, the EpsY/EpsV complex could, thus, have a height of ~160 to 240 Å across the periplasm, supporting that they can jointly span the periplasm.

10.1128/mbio.02032-22.7 FIG S7 AlphaFold models of EpsV, ExoC, and WzcB. (A to C) pLDDT and pAE plots for five models of the indicated proteins as predicted by AlphaFold. For all three proteins, model rank 1 (marked by a green box) was used for further analysis and is shown colored based on the pLDDT values. In the lower left of panels B and C, the proteins are shown in orange and arrows indicate the lengths of the extended α-helical stretches. Middle panels, superimposition of a protomer from the solved octameric structure of Wzc (PDB 7NHR) (30) and the relevant protein model. ExoC aligns to Wzc with an RMSD of 2.706Å over 776 C*_α_*, and WzcB aligns to Wzc with an RMSD of 3.946Å over 1,719 C*_α_*. Download FIG S7, PDF file, 2.0 MB. Copyright © 2022 Schwabe et al. 2022 Schwabe et al. https://creativecommons.org/licenses/by/4.0/ This content is distributed under the terms of the Creative Commons Attribution 4.0 International license.

Similarly, monomeric ExoC of the SPS pathway and WzcB of the BPS pathway have long α-helical periplasmic regions in high-accuracy AlphaFold models (Fig. S7B and C). Together, these *in silico* analyses support the idea that ^D1D2^OPX proteins function together with a PCP with an extended periplasmic part.

### Coupled genes for short periplasmic OPX proteins and OM β-barrel proteins are widespread in Gram-negative bacteria.

Forty-one of the 48 myxobacterial genes for ^D1D2^OPX proteins are syntenic with a gene encoding an 18-stranded β-barrel protein (Fig. S1A and S6). We took advantage of this observation to assess bioinformatically how widespread short, periplasmic OPX proteins are and whether they are coupled with an OM β-barrel protein. Specifically, we identified OPX candidates in 6,607 fully sequenced prokaryotic genomes across 49 phyla using the PES motif (Pfam PF02563) as described previously (6). To substantiate that the identified proteins are part of a polysaccharide biosynthesis pathway, we included only OPX candidate genes with a polysaccharide biosynthesis gene within five genes upstream or downstream (see Materials and Methods). In total, we identified 4,257 OPX proteins and subsequently used 2,749 representative proteins to determine whether they have a C-terminal α-helix and/or are coupled to an OM β-barrel protein (see Materials and Methods). To determine the coupling of OPX proteins and OM β-barrel proteins, we searched for genes encoding OM β-barrel proteins within five genes of a gene encoding an OPX protein (see Materials and Methods). After verifying the β-barrel fold and OM localization computationally (see Materials and Methods), we identified 486 such OM β-barrel proteins.

**FIG 4:**
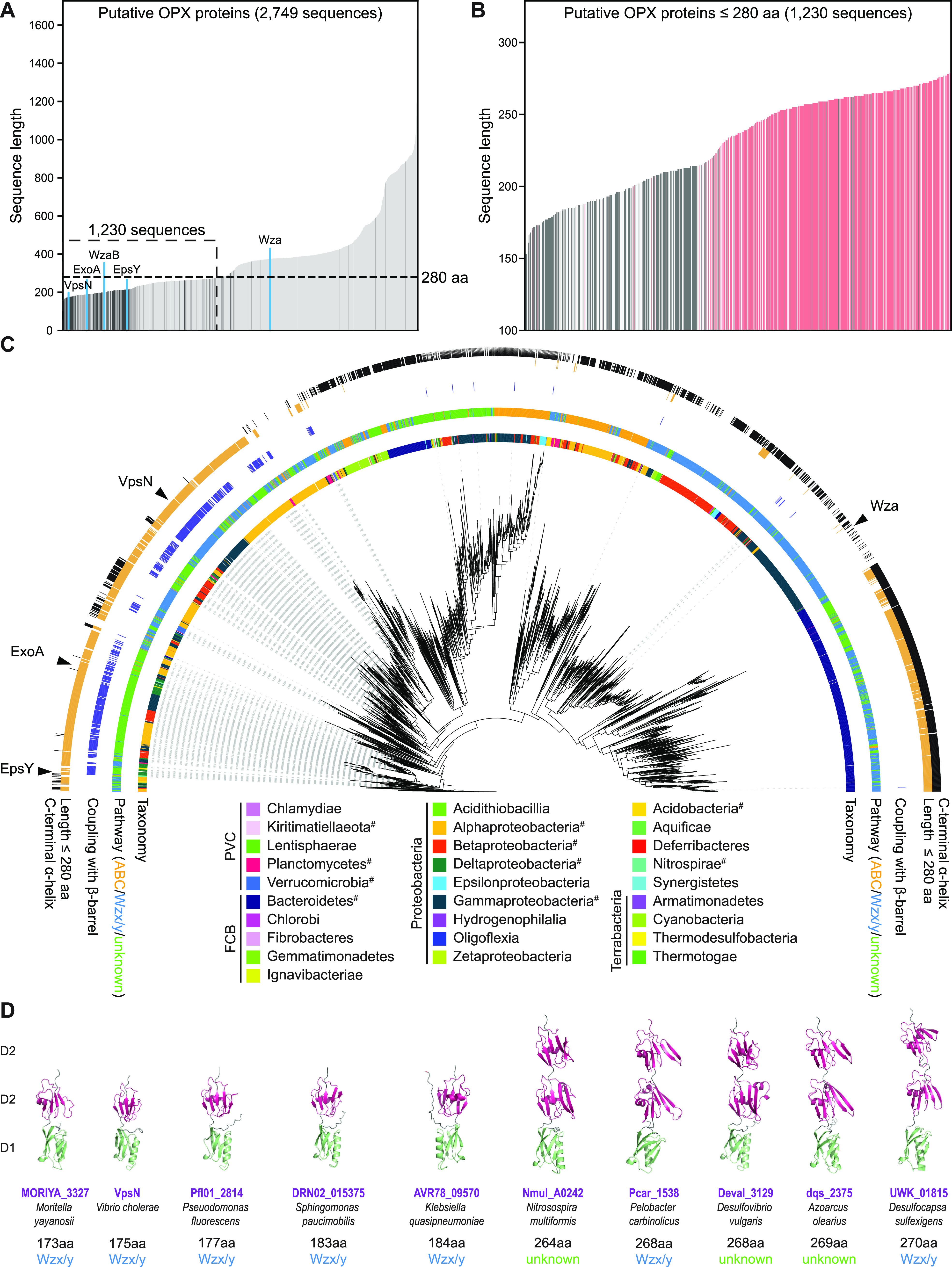
Computational genomics of OPX proteins. (A) Length distribution of 2,749 OPX proteins. OPX proteins encoded within a distance of five genes of a gene encoding a β-barrel protein are shown in dark gray, and the remaining OPX proteins are in light gray. OPX proteins discussed in the text are highlighted in blue. The 1,230 short OPX proteins are indicated based on the upper size limit of ≤280 aa. (B) Length distribution of OPX proteins of ≤280 aa. Four hundred fifty-three OPX proteins with a coupled β-barrel protein and no C-terminal α-helix are in dark gray; 535 OPX proteins with no coupled β-barrel protein but a C-terminal α-helix are in red. Proteins not matching these criteria are in light gray. (C) Maximum-likelihood phylogenetic tree built from the PES motif of the 2,749 OPX proteins. The inner ring is colored based on NCBI taxonomy (https://www.ncbi.nlm.nih.gov/taxonomy/). PVC, FCB, and Terrabacteria are superphyla, and the phylum *Proteobacteria* is divided into classes. #, this phylum contains an OPX protein(s) of ≤280 aa coupled to a β-barrel protein(s). The second ring indicates the pathway assigned to individual OPX proteins. The third ring indicates, in dark blue, coupling with a β-barrel protein. The fourth ring indicates, in orange, OPX proteins of ≤280 aa. The fifth ring indicates, in black, whether an OPX protein has a C-terminal α-helix. (D) AlphaFold models of indicated OPX proteins. Domains are labeled D1 and D2 and colored according to the homologous domains in Wza (Fig. 2A). Model rank 1 is shown for all structures.

The 2,749 OPX proteins range in length from 138 to 1,728 aa (Fig. 4A). Interestingly, the length distribution of the 486 OPX proteins with a coupled β-barrel protein is highly skewed toward shorter OPX proteins (Fig. 4A and B). Based on the size distribution of OPX proteins with a coupled β-barrel protein, and because EpsY, ExoA, and WzaB are 217 aa, 190 aa, and 204 aa, respectively, we empirically defined a size cutoff for short OPX proteins of 280 aa (Fig. 4A and B). We built a phylogenetic tree of all 2,749 OPX proteins based on their PES motif and found these proteins in 28 phyla (Fig. 4C). We then located the branches of the tree that correspond to OPX proteins with a size of ≤280 aa and found that those with a coupled β-barrel protein are present in 10 phyla (Fig. 4C).

A total of 1,230 OPX proteins are ≤280 aa, and among these, 453 proteins have a coupled β-barrel protein (Fig. 4B and C). Conversely, 93% of the β-barrel proteins are coupled to an OPX protein of ≤280 aa. Interestingly, among the 777 OPX proteins of ≤280 aa and without a coupled β-barrel protein, 535 have a C-terminal α-helix (Fig. 4B and C) and are found mostly in the *Bacteroidetes* (Fig. 4C). Thus, generally, we observe a dichotomy among the 1,230 OPX proteins of ≤280 aa. Typically, these OPX proteins have either a coupled β-barrel protein and no C-terminal α-helix (453 proteins) or no coupled β-barrel protein but a C-terminal α-helix (535 proteins). For the latter group of OPX proteins, we suggest that they likely function similarly to Wza.

Focusing on the 453 OPX proteins of ≤280 aa with a coupled β-barrel protein and no C-terminal α-helix, we found, based on genomic context, that 185 are part of a Wzx/Wzy-dependent pathway, 14 are part of an ABC transporter-dependent pathway, and 287 were not assigned to a pathway because the closest polysaccharide biosynthesis gene encodes a PCP and some of these proteins can be difficult to assign to a specific type of pathway (6). Among the short OPX/β-barrel couples identified, we found VpsN/VpsM of Vibrio cholerae (Fig. 4A, C, and D). These two proteins are important for *Vibrio* polysaccharide (VPS) synthesis, biofilm formation, and intestinal colonization in a mouse model (31), thus validating the outlined computational approach. Analysis of the domain architecture of the short OPX proteins is difficult because, except for the PES motif in the D1 domain, their sequences are not well conserved. Therefore, to analyze the domain architecture of the 453 short OPX proteins with a coupled β-barrel protein, we analyzed the domain structure of five proteins of ≤200 aa and five proteins of >250 aa. Intriguingly, the five proteins with a length of <200 aa all had the ^D1D2^OPX architecture, while the proteins of >250 aa had a ^D1D2D2^OPX architecture (Fig. 4D; Fig. S8).

10.1128/mbio.02032-22.8 FIG S8 AlphaFold models of the indicated proteins. pLDDT and pAE plots for five models of the indicated proteins predicted by AlphaFold. For all 10 proteins, model rank 1 (marked by a green box) was used for further analysis and is shown colored based on the pLDDT values indicated at the bottom of the figure. Signal peptides were removed before generating a model. Download FIG S8, PDF file, 1.1 MB. Copyright © 2022 Schwabe et al. 2022 Schwabe et al. https://creativecommons.org/licenses/by/4.0/ This content is distributed under the terms of the Creative Commons Attribution 4.0 International license.

We conclude that the coupling between ^D1D2^OPX proteins and OM β-barrel proteins is conserved and widespread in Gram-negative bacteria and that this coupling can be extended to include the ^D1D2D2^OPX variants.

### Conclusions.

**FIG 5:**
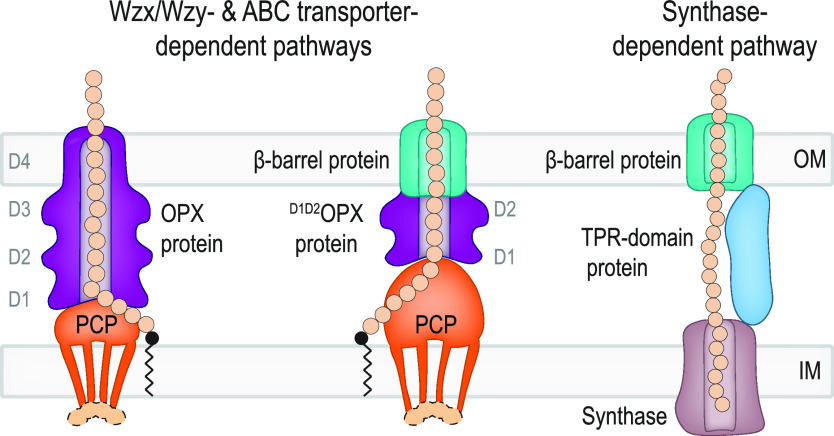
Three different mechanisms for polysaccharide export across the OM. Left schematic, in classical Wzx/Wzy- and ABC transporter-dependent pathways, polysaccharide transfer across the periplasm and OM is mediated by a complex composed of a PCP and an OPX protein with an α-helical barrel in the OM. Middle schematic, in pathways in which the OPX protein lacks domain D4 (^D1D2^OPX or ^D1D2D2^OPX proteins), a β-barrel protein constitutes the OM part of the composite OPX/β-barrel protein translocon. The D1 to D4 and D1 to D2 rings of the OPX proteins are indicated. In Wzx/Wzy-dependent pathways, the PCP can be associated with a cytoplasmic BY kinase (light orange). Synthesis, polymerization, and translocation of the polysaccharide across the IM are not shown. Right schematic, in synthase-dependent pathways, transfer across the periplasm and the OM depends on a TPR domain-containing protein and a β-barrel protein translocon in the OM.

Here, using experimental approaches and computational analyses, we provide evidence that polysaccharides can be exported across the OM via a composite periplasmic OPX protein/OM β-barrel protein translocon (Fig. 5). The domain architecture of the periplasmic OPX proteins can be ^D1D2^OPX or ^D1D2D2^OPX, giving rise to ^D1D2^OPX/β-barrel translocons and ^D1D2D2^OPX/β-barrel translocons. The ^D1D2^OPX/β-barrel translocons likely function with a PCP with extra-long periplasmic α-helical regions to span the periplasm and compensate for the short OPX protein (Fig. 5). These systems are widespread in Gram-negative bacteria and add to the two established mechanisms for OM export, i.e., the Wza-like OPX protein in Wzx/Wzy- and ABC transporter-dependent pathways and the OM 16- to 18-stranded β-barrel protein in synthase-dependent pathways (Fig. 5). This work provides a framework for future biochemical and structural studies of EpsX and EpsY as well as of other polysaccharide biosynthesis pathways containing a ^D1D2^OPX or ^D1D2D2^OPX protein together with a coupled OM β-barrel protein.

The existence of three systems for polysaccharide export across the OM poses the question of their evolutionary origin. Our current analysis does not suggest an evolutionary scenario for their emergence, and this will likely depend on detailed studies of the taxonomic distribution of the three systems.

During the preparation of this article, Saïdi et al. (20) independently identified EpsX, ExoB, and WzpB as cooccurring with EpsY, ExoA, and WzaB and suggested that these proteins could make up a translocon for export of polysaccharides across the OM, thus independently supporting our findings.

## MATERIALS AND METHODS

### Strains and cell growth.

All M. xanthus strains are derivatives of the WT strain DK1622 (32) and are listed in Table 1. The plasmids and oligonucleotides used are listed in Table 2 and in Table S1 in the supplemental material. In-frame deletions were generated as described previously (33). The plasmids for complementation experiments were integrated in a single copy by site-specific recombination into the Mx8 *attB* site and the plasmid for ectopic expression of *sfGFP-FLAG* into the *18-19* site. All plasmids were verified by DNA sequencing, and all strains were verified by PCR. M. xanthus was grown at 32°C in 1% CTT broth (1% [wt/vol] Bacto casitone, 10 mM Tris-HCl [pH 8.0], 1 mM K_2_HPO_4_/KH_2_PO_4_ [pH 7.6], 8 mM MgSO_4_) or on 1.5% agar supplemented with 1% CTT and kanamycin (50 μg mL^−1^) or oxytetracycline (10 μg mL^−1^) when required (34). Plasmids were propagated in E. coli Mach1 at 37°C in lysogeny broth (LB) (35) supplemented with kanamycin (50 μg mL^−1^) or tetracycline (20 μg mL^−1^).

**TABLE 1:** Strains used in this work

Species and strain	Genotype	Reference or source
M. xanthus		
DK1622	WT	32
DK10410	Δ*pilA*	67
SA3922	Δ*gltB*	23
SA7400	Δ*epsZ*	17
SA7406	Δ*epsV*	17
SA7408	Δ*epsY*	17
SA7412	Δ*epsY attB*::pMP030 (P*_pilA_ epsY*)	17
SA11550	Δ*epsX*	This study
SA11554	Δ*epsX attB*::JSc007 (P*_pilA_ epsX*)	This study
SA11592	Δ*epsY attB*::pJSc053 (P*_pilA_ epsY-FLAG*)	This study
SA11597	Δ*epsY attB*::pMP030 (P*_pilA_ epsY*) *18-19*::pJSc056 (P*_pilA_ sfGFP-FLAG*)	This study
		
E. coli Mach1	Δ*recA1398 endA1 tonA* Φ80Δ*lacM15* Δ*lacX74 hsdR*(r_K_^−^ m_K_^+^)	Invitrogen

**TABLE 2:** Plasmids used in this work

Plasmid	Description	Reference
pBJ114	Km^r^ *galK*	68
pSW105	Km^r^ P*_pilA_*	69
pDS75	Tet^r^ P*_pilA_*	36
pJSc004	pBJ114, in-frame deletion construct for *epsX* (*MXAN_7418*), Km^r^	This study
pJSc007	pSW105, complementation construct for *epsX* (*MXAN_7418*) expressed from the *pilA* promoter, Km^r^	This study
pJSc053	pSW105, construct for *epsY-FLAG* expression from the *pilA* promoter, Km^r^	This study
pJSc056	pSWU30 for *18-19* site, construct for *sfGFP-FLAG* expression from the *pilA* promoter, Tet^r^	This study

10.1128/mbio.02032-22.9 TABLE S1 Oligonucleotides used in this work. Download Table S1, DOCX file, 0.01 MB. Copyright © 2022 Schwabe et al. 2022 Schwabe et al. https://creativecommons.org/licenses/by/4.0/ This content is distributed under the terms of the Creative Commons Attribution 4.0 International license.

### Plasmid construction.

For pJSc004 (for generation of in-frame deletion of *epsX*), up- and downstream fragments were amplified from genomic DNA of DK1622 using the primer pairs 7418-A/7418-B and 7418-C/7418-D, respectively. Subsequently, the AB and CD fragments were used as templates for an overlapping PCR with the primer pair 7418-A/7418-D to generate the AD fragment. The AD fragment was digested with KpnI/XbaI and cloned in pBJ114. For pJSc007 (for generation of a strain ectopically expressing *epsX* from the *pilA* promoter), *epsX* was amplified from genomic DNA of DK1622 using 7418-P*pilA*-for and 7418-Pnat/P*pilA-*rev. Subsequently, the PCR fragment was digested with XbaI/HindIII and cloned into pSW105. For pJSc053 (for generation of a strain ectopically expressing *epsY-FLAG* [FLAG tag fused after aa 43 of EpsY] from the *pilA* promoter), fragment 1 was amplified from genomic DNA using epsY-PpilA-for and epsY-Flag43 rev. Fragment 2 was amplified from genomic DNA using epsY-PpilA rev and epsY-Flag43-for. Fragments 1 and 2 were used as templates for an overlapping PCR with the primer pair epsY-PpilA-for and epsY-PpilA-rev. Subsequently, the PCR fragment was digested with XbaI/HindIII and cloned into pSW105. For pJSc056 (for generation of a strain ectopically expressing *sfGFP-FLAG* from the *pilA* promoter, for integration into the *18–19* site), a fragment containing *sfGFP-FLAG* under the control of the *pilA* promoter was obtained from pMAT219 (25) using EcoRI/HindIII restriction sites and cloned into pDS75 (36) (a derivate of pSWU30, where the Mx8 *attB* locus was exchanged with the *MXAN_18-19* intergenic region) after digesting it with EcoRI/HindIII to remove the insert.

### Detection of EPS.

Colony-based EPS assays were performed as described previously (17). Briefly, exponentially growing cells were harvested (3 min, 6,000 × *g* at room temperature [RT]) and resuspended in 1% CTT to a calculated density of 7 × 10^9^ cells mL^−1^, and 20-μL aliquots were placed on 0.5% agar plates supplemented with 0.5% CTT and 10 or 20 μg mL^−1^ of trypan blue or Congo red, respectively. Plates were incubated at 32°C and imaged at 24 h.

### Motility assays.

Motility assays were performed as described previously (37). Briefly, exponentially growing cells were harvested (3 min, 6,000 × *g*, RT) and resuspended in 1% CTT to a density of 7 × 10^9^ cells mL^−1^. Five-microliter volumes of cell suspensions were placed on 0.5% and 1.5% agar (Invitrogen) supplemented with 0.5% CTT and incubated at 32°C for 24 h. Cells were imaged using a M205FA stereomicroscope (Leica) and a DMi8 inverted microscope (Leica) equipped with a Hamamatsu ORCA-Flash V2 digital CMOS camera (Hamamatsu Photonics) and DFC9000 GT camera (Leica), respectively.

### Immunoblots.

Immunoblotting was performed as described previously (35). For sample preparation, M. xanthus cells were harvested from exponentially growing suspension cultures and resuspended in SDS lysis buffer. Proteins were loaded from an equal number of cells per sample. As primary antibodies, rabbit polyclonal anti-FLAG (1:2,000; Rockland) and anti-PilC (1:2,000) (38) antibodies were used, with horseradish peroxidase (HRP)-conjugated anti-rabbit immunoglobulin G (1:15,000, Sigma) as the secondary antibody. Immunoblots were developed using Immobilon Forte Western HRP substrate (Millipore) on a LAS-4000 imager (Fujifilm).

### RT-qPCR.

Total RNA from M. xanthus cells grown on 1.5% agar supplemented with 1% CTT was extracted using the Monarch total RNA miniprep kit (New England Biolabs [NEB]). Briefly, 10^9^ cells were scraped off the agar plates, resuspended in 200 μL lysis buffer (100 mM Tris-HCl [pH 7.6], 1 mg mL^−1^ lysozyme), and incubated at 25°C for 5 min. RNA purification was performed according to the manufacturer's protocol. DNA was removed using Turbo DNase (Thermo Fisher Scientific), and DNase was removed by using the Monarch RNA cleanup kit (50 μg; NEB). A LunaScript RT supermix kit (NEB) was used to generate cDNA using 1 μg RNA. qPCRs were performed on an Applied Biosystems 7500 real-time PCR system using the Luna universal qPCR mastermix (NEB) with the primers listed in Table S1. Data analysis was performed using the comparative threshold cycle (*C_T_*) method (39). *MXAN_3298*, encoding the elongation factor Tu, served as an internal reference gene as described previously (26).

### Proteomic analysis using DIA-MS.

The total proteome of M. xanthus cells grown on 1% CTT–1.5% agar plates was determined as described previously (25). Peptide mixtures were then analyzed using liquid chromatography-mass spectrometry (LC-MS) on an Exploris 480 instrument connected to an Ultimate 3000 RSLCnano system with a ProFlow upgrade and a Nanospray Flex ion source (all Thermo Scientific). Peptide separation was performed on a reverse-phase high-performance liquid chromatography (HPLC) column (75 μm by 42 cm) packed in-house with C_18_ resin (2.4 μm; Dr. Maisch). The following separating gradient was used: 94% solvent A (0.15% formic acid) and 6% solvent B (99.85% acetonitrile, 0.15% formic acid) to 25% solvent B over 95 min and to 35% B for additional 25 min at a flow rate of 300 nL min^−1^. The data-independent acquisition–mass spectrometry (DIA-MS) acquisition method was adapted from reference 40. Briefly, the spray voltage was set to 2.0 kV, the funnel radio frequency level was set at 55, and the heated capillary temperature was set at 275°C. For DIA experiments, full MS resolutions were set to 120.000 at *m/z* 200 with an automatic gain control (AGC) target of 300% and a maximum injection time (IT) of 50 ms. The mass range was set at 350 to 1,400. The AGC target value for fragment spectra was set at 3000%. Forty-nine windows of 15 Da were used with an overlap of 1 Da. Resolution was set to 15,000, and IT was set to 22 ms. A stepped high-energy collisional dissociation (HCD) collision energy of 25, 27.5, and 30% was used. MS1 data were acquired in profile, and MS2 DIA data were acquired in centroid mode.

Analysis of DIA data was performed using DIA-NN version 1.8 (41) with a UniProt protein database for M. xanthus. Full tryptic digestion was allowed with two missed cleavage sites and with oxidized methionines (variable) and carbamidomethylated cysteines (fixed). “Match between runs” and “remove likely interferences” options were enabled. The neural network classifier was set to the single-pass mode, and protein inference was based on “genes.” The quantification strategy was set to any LC (high accuracy). Cross-run normalization was set to “RT-dependent.” Library generation was set to “smart profiling.” The DIA-NN “report” output was used to sum the unique peptide intensities, and identified proteins were filtered out if *q* values (false discovery rates) exceeded 0.01. Protein intensities were normalized with the cyclic loess method using the R package NormalyzerDE (42).

Pulldown experiments were performed as described previously (25). Briefly, M. xanthus cells grown on 1% CTT–1.5% agar plates were collected (80 mg per replicate) and washed in 1 mL phosphate-buffered saline (PBS) (137 mM NaCl, 2.7 mM KCl, 10 mM Na_2_HPO_4_, 1.8 mM KH_2_PO_4_, pH 7.5). Cells were resuspended in 150 μL detergent lysis solution consisting of 8% CHAPS{3-[(3-cholamidopropyl)dimethylammonio]-1-propanesulfonate hydrate (wt/vol)), 8% 3-(*N,N*-dimethylmyristylammonio)propanesulfonate (wt/vol), 8% sodium lauroyl sarcosinate (SLS) (wt/vol), 0.1% Nonidet P-40 (NP-40) (vol/vol), 40% glycerol (vol/vol)), 150 μL PBS, and 33 μL protease inhibitor concentrate (1 tablet protease inhibitor dissolved in 1 mL PBS; Roche). Lysis was performed for 10 min at RT. The total volume was increased to 5 mL with PBS, 5 μL Benzonase (Merck) was added, and incubation proceeded under rotation at 4°C for 60 min. Cell debris was removed by centrifugation (15 min, 10,000 × *g* at 4°C). Fifteen microliters of equilibrated magnetic anti-FLAG M2 beads (Sigma) was added to the cleared supernatants and incubated with rotation at 4°C for 60 min. Beads were collected using a magnetic separator and washed twice with 700 μL PBS-detergent wash buffer (detergent lysis solution diluted 1:30 in PBS) and five times with 100 mM ammonium bicarbonate (Sigma-Aldrich) to remove detergents. Protein elution from beads was carried out at 90°C for 10 min in the presence of 2% SLS (wt/vol) in 100 mM ammonium bicarbonate. The eluate was reduced and alkylated and subsequently acetone precipitated using an 8× volume excess of ice-cold acetone, followed by incubation at −20°C overnight. After centrifugation (10 min, 10,000 × *g*), the protein pellet was washed twice with 200 μL cold methanol and air dried. Proteins were reconstituted in 100 mM ammonium bicarbonate and digested in the presence of 1 μg trypsin (Serva). Postdigestion, tryptic peptides were desalted using C_18_ solid-phase extraction (see reference 25) and analyzed via LC-MS using data-independent acquisition (see above) with the exception that the separating gradient length was reduced to 30 min and DIA raw data were analyzed with DIA-NN with the above-described setting and further evaluated in SafeQuant (43) using median protein intensities for normalization, background value imputation from normal distribution (width, 0.3; down shift, 1.8), and moderate eBayes *t*-test statistics as a significance strategy (44). For calculation of enrichment factors in samples over that of the control, identified proteins were filtered out if the peptide count in at least one sample replicate fell below 3. Proteins with an absolute abundance difference of ≥8 (log_2_-fold enrichment of ≥3) in samples over that of the control and a *P* value of ≤0.001 (−log_10_ *P* value ≥ 3) were considered enriched.

### Bioinformatics.

The phylogenetic tree of myxobacteria was prepared as described previously (45) in MEGA-X (46) using the neighbor-joining method (47) and the genome sequences listed in Table S2.

10.1128/mbio.02032-22.10 TABLE S2 Fully sequenced myxobacterial genomes used for the 16S RNA tree. Download Table S2, DOCX file, 0.01 MB. Copyright © 2022 Schwabe et al. 2022 Schwabe et al. https://creativecommons.org/licenses/by/4.0/ This content is distributed under the terms of the Creative Commons Attribution 4.0 International license.

To illustrate the operon structure of the *eps* loci, transcriptome sequencing (RNA-seq) and cappable-seq data from reference 48 were used.

Full-length protein sequences or sequences in which the signal peptide was identified with SignalP 6.0 (49) and removed were used for AlphaFold and AlphaFold-Multimer modeling via ColabFold (50–52) using the Alphafold2_mmseqs2 notebook with default settings, except that recycles were set to six. Predicted local distance difference test (pLDDT) and predicted alignment error (pAE) graphs of the five models generated by the Alphafold2_mmseqs2 notebook were made using a custom-made Matlab R2020a (The MathWorks) script. Ranking of the models was performed based on combined pLDDT and pAE values, with the best-ranked models used for further analysis and presentation. Per-residue model accuracy was estimated based on pLDDT values (>90, high accuracy; 70 to 90, generally good accuracy; 50 to 70, low accuracy; <50, should not be interpreted) (50). Relative domain positions were validated by pAE. The pAE graphs indicate the expected position error at residue X if the predicted and true structures were aligned on residue Y; the lower the pAE value, the higher the accuracy of the relative position of residue pairs and, consequently, the relative position of domains/subunits/proteins (50). PyMOL version 2.4.1 (http://www.pymol.org/pymol) was used to analyze and visualize the models. Structural alignments were performed using the PyMOL Alignment plugin with default settings. For calculation of surface charges, protein models were prepared using pdb2pqr (53) and electrostatics were calculated via the Adaptive Poisson-Boltzmann Solver (APBS) (53) plugin in PyMOL with default settings. Foldseek was used to identify protein homologs of EpsX in the PDB 100 database (54).

For the cooccurrence analysis of OPX and OM β-barrel proteins, we generated a database of 6,607 fully sequenced prokaryotic genomes that mirrors the KEGG database (55) and comprehensively covers all major prokaryotic phyla. OPX proteins encoded in these genomes were identified with HMMsearch of the HMMER package (v3.3) (56) using the Pfam (57) domain Poly_export (PF02563) with the Pfam gathering threshold.

Subsequently, we generated a database corresponding to the proteins encoded by five genes up- and downstream of the identified OPX proteins. This database was searched for the Pfam domains of polysaccharide synthesis proteins (ABC2_membrane [PF01061], ABC_tran [PF00005], Polysacc_synt [PF01943], Polysacc_synt3 [PF13440], Wzt_C [PF14524], Wzy_C [PF04932], and Wzz [PF02706]) to (i) corroborate that the OPX protein is encoded in a polysaccharide synthesis gene cluster and (ii) assign a pathway to the OPX protein. PCPs identified with the Wzz domain were additionally analyzed for the presence of the tyrosine kinase domain to assign them as PCP-2. OPX proteins that were not encoded in a polysaccharide synthesis gene cluster were excluded from further analysis. To remove redundancy in the data set, OPX protein sequences were clustered to a 90% identity threshold using Cd-hit (58) and further analysis was carried out on the resulting set of representative sequences.

To generate the phylogenetic tree of the OPX protein sequences, the sequence matching the Poly_export domain of the OPX proteins was aligned using MUSCLE (59) and a maximum likelihood tree was inferred using IQ-Tree (60) with automated model selection and support calculated with 1,000 ultrafast bootstrap replicates. The tree was visualized and annotated using iTOL (61).

The secondary structure of the OPX proteins was determined using S4pred (62) and then scanned for an α-helix within the last 20 C-terminal residues. C-terminal α-helices were considered positive if they extended over more than nine residues and did not contain more than one gap of two residues predicted to be nonhelical.

β-Barrel proteins were initially searched for by using hmmbuild to build a hidden Markov model (HMM) from an alignment of 31 EpsX, ExoB, and MXAN_1916 homologs generated with MUSCLE. In a second step, proteins from the KEGG orthology group VpsM (K20920) were aligned and used to build a second HMM. Both models were used to search the proteins encoded by five genes up- and downstream of the genes encoding OPX proteins. β-Barrel fold and OM localization of identified proteins was verified using PRED-TMBB2 (63) and PSORTb 3.0 (64). β-Barrel proteins with fewer than 16 β-strands were classified as false positive and were not considered further.

### Statistics.

The Welch’s *t* test was performed to determine the statistical differences between the samples.

### Data availability.

The data that support the findings of this study are included in the article or in the supplemental material. The proteomics and pulldown data have been deposited in the ProteomeXchange Consortium via the PRIDE (65) partner repository with the data set identifier PXD035138.
