# Supplementary figures and images for: Evidence for a Widespread Third System for Bacterial Polysaccharide Export across the Outer Membrane Comprising a Composite OPX/β-Barrel Translocon
Source: mBio. 2022 Aug 16;13(5):e02032-22. doi: 10.1128/mbio.02032-22 (PMC9601211; doi:10.1128/mbio.02032-22)

A

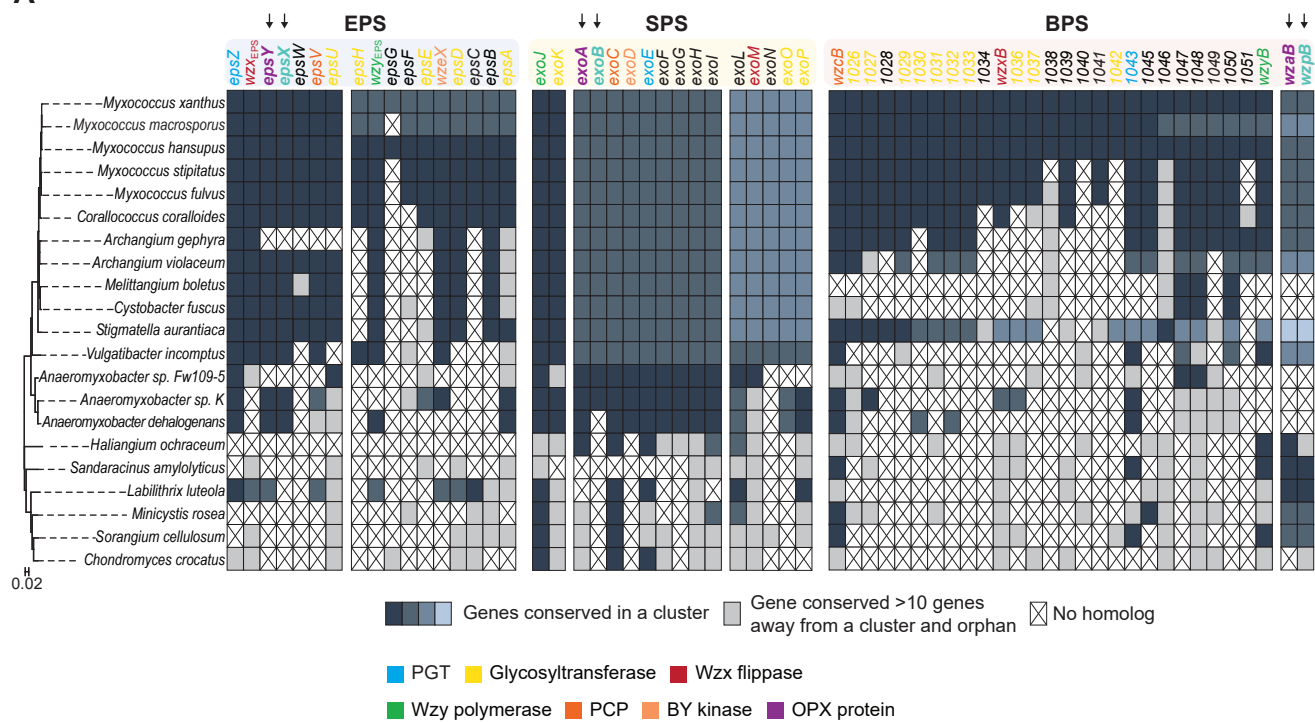

B

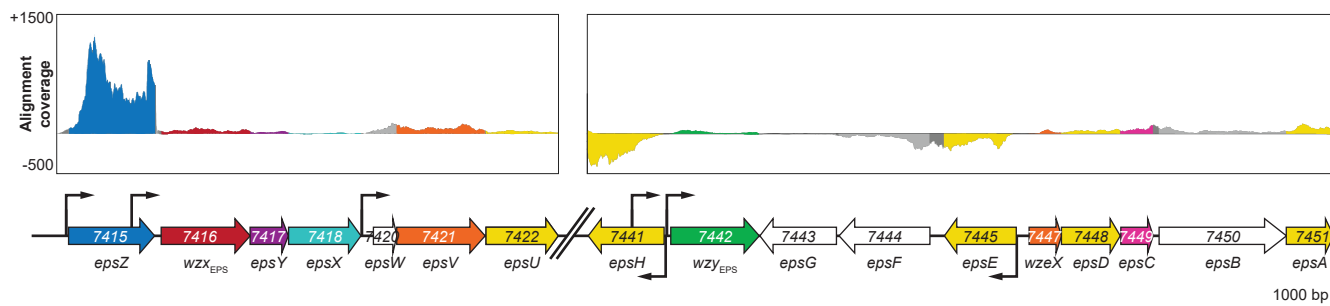

Supplement: FIG S1 [file mbio.02032-22-s0001.pdf]

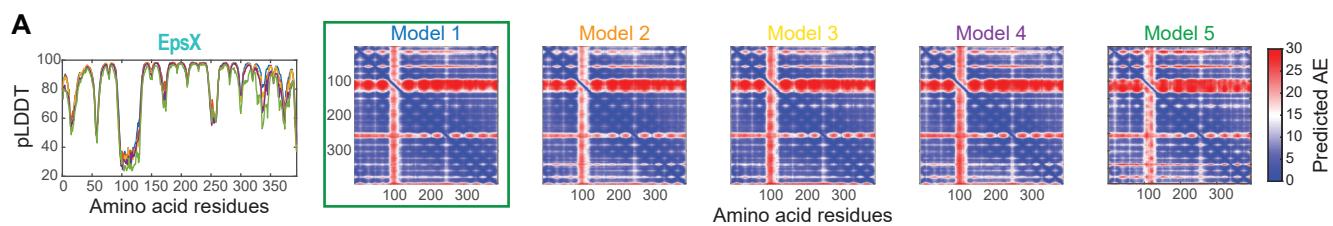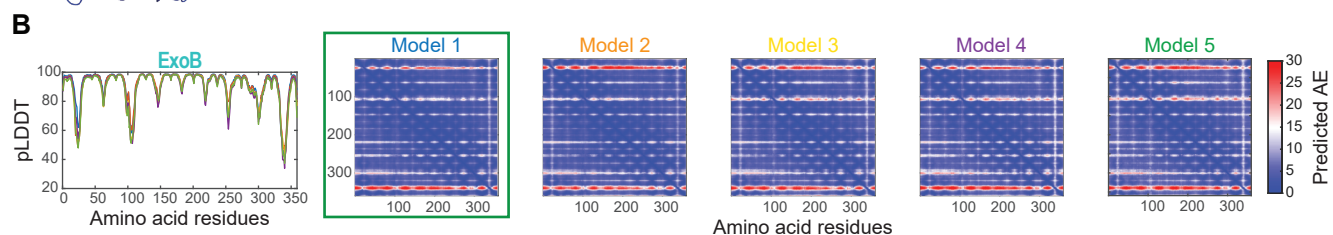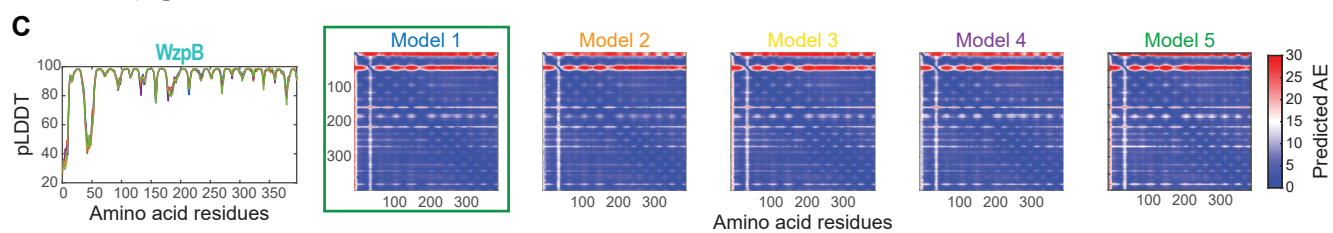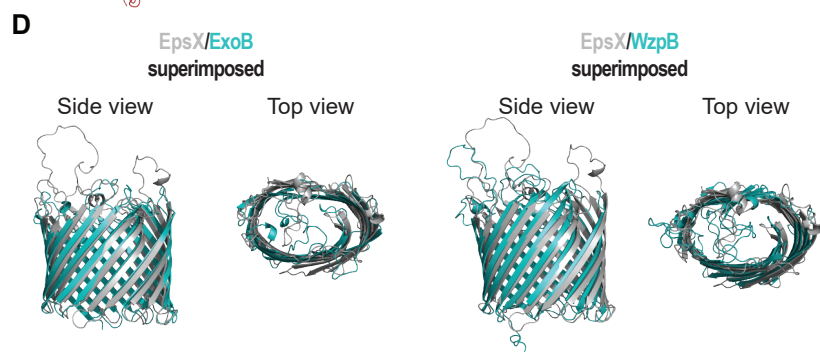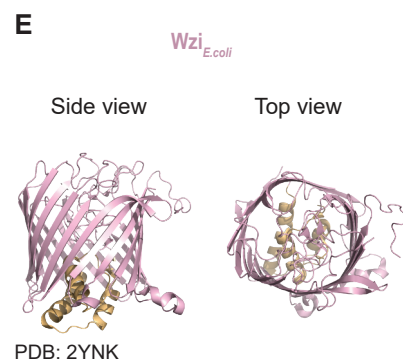

Supplement: FIG S2 [file mbio.02032-22-s0002.pdf]

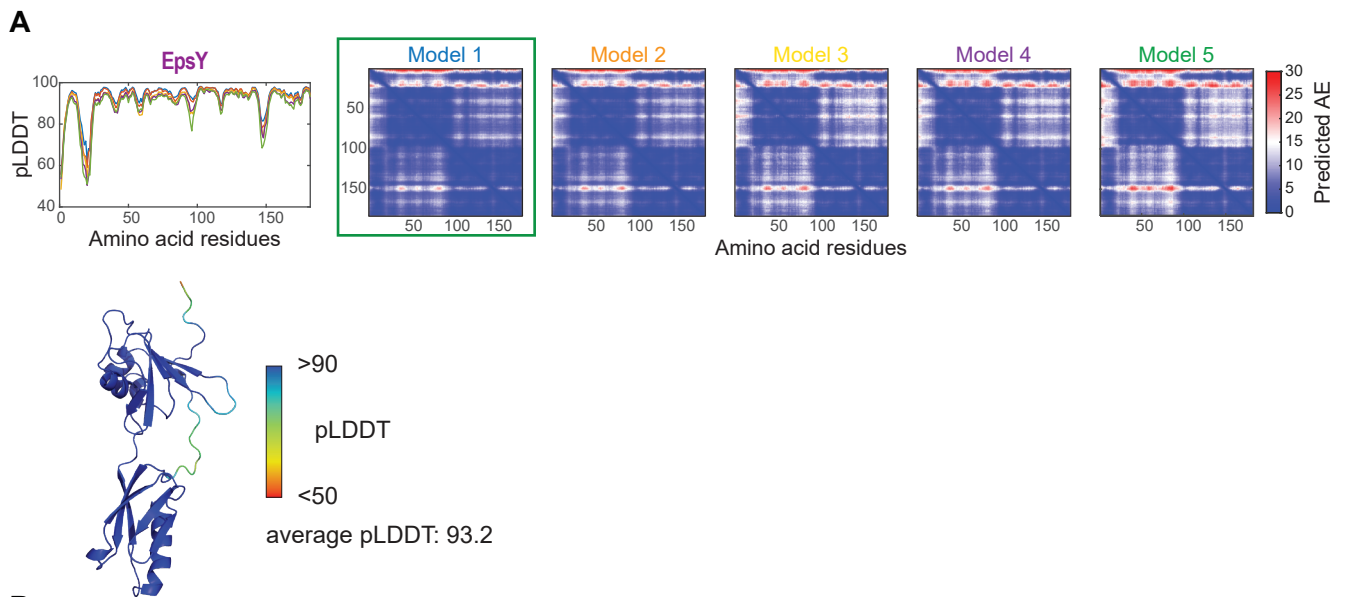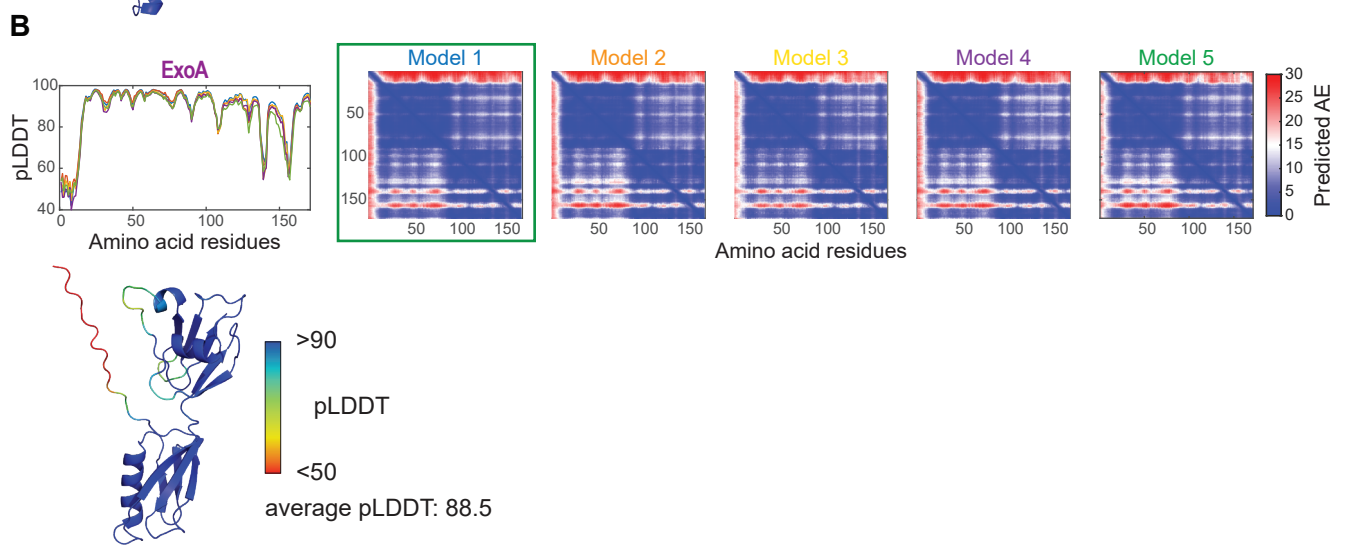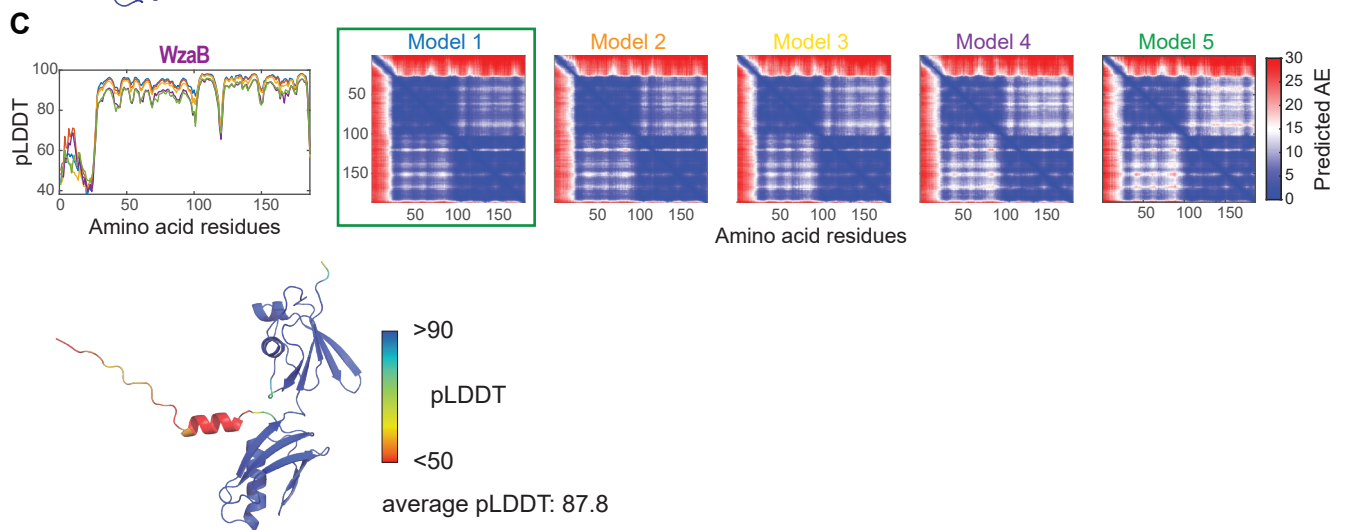

Supplement: FIG S4 [file mbio.02032-22-s0004.pdf]

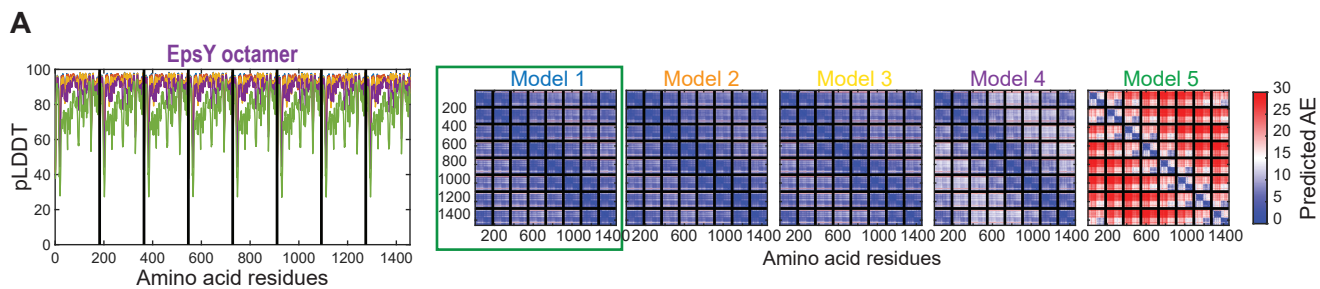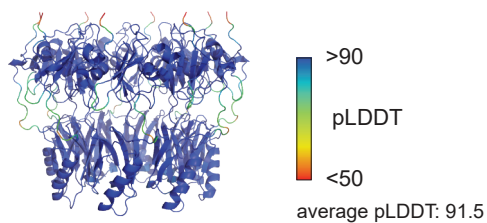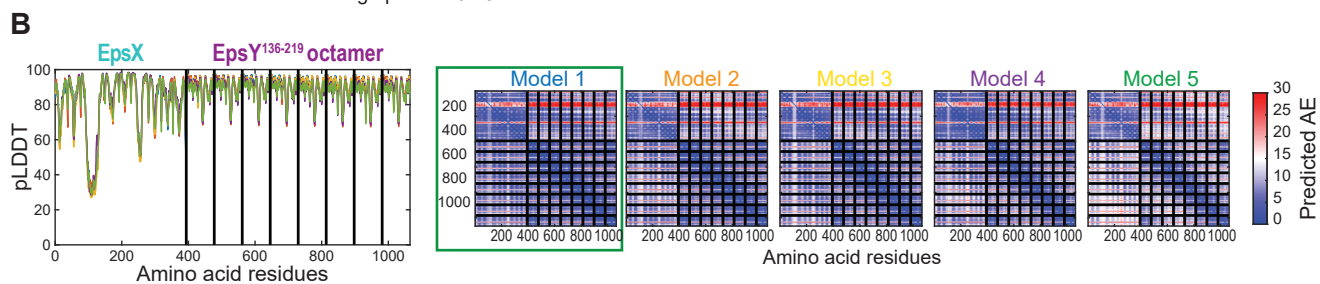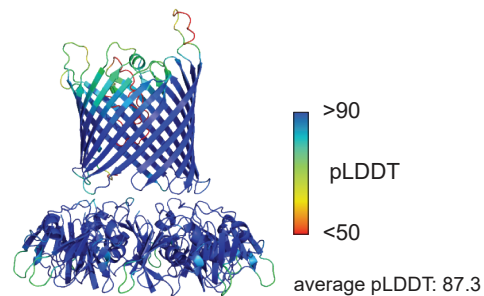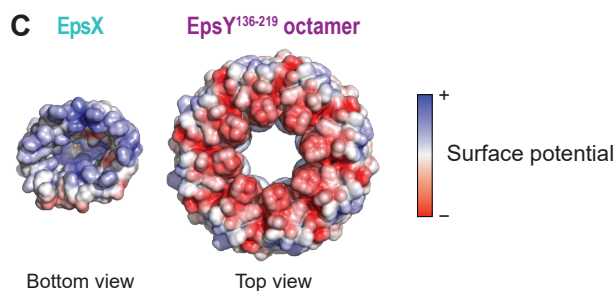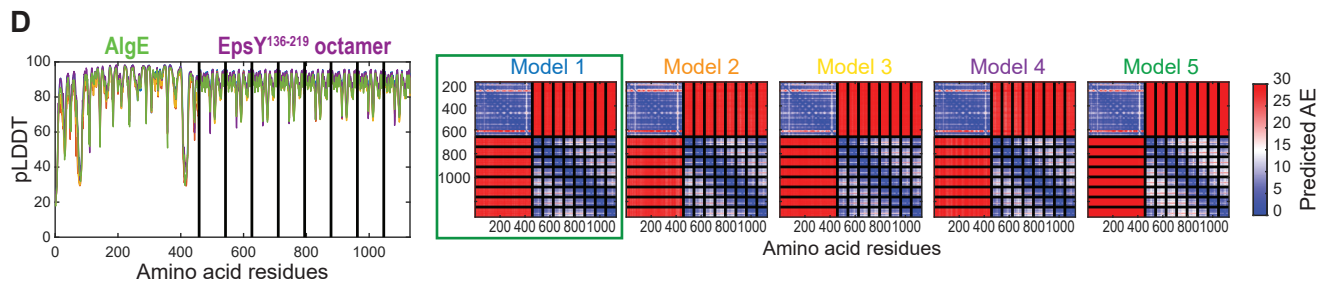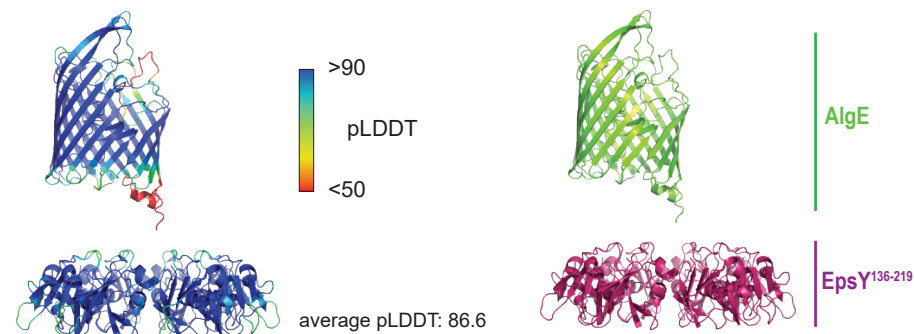

Supplement: FIG S5 [file mbio.02032-22-s0005.pdf]

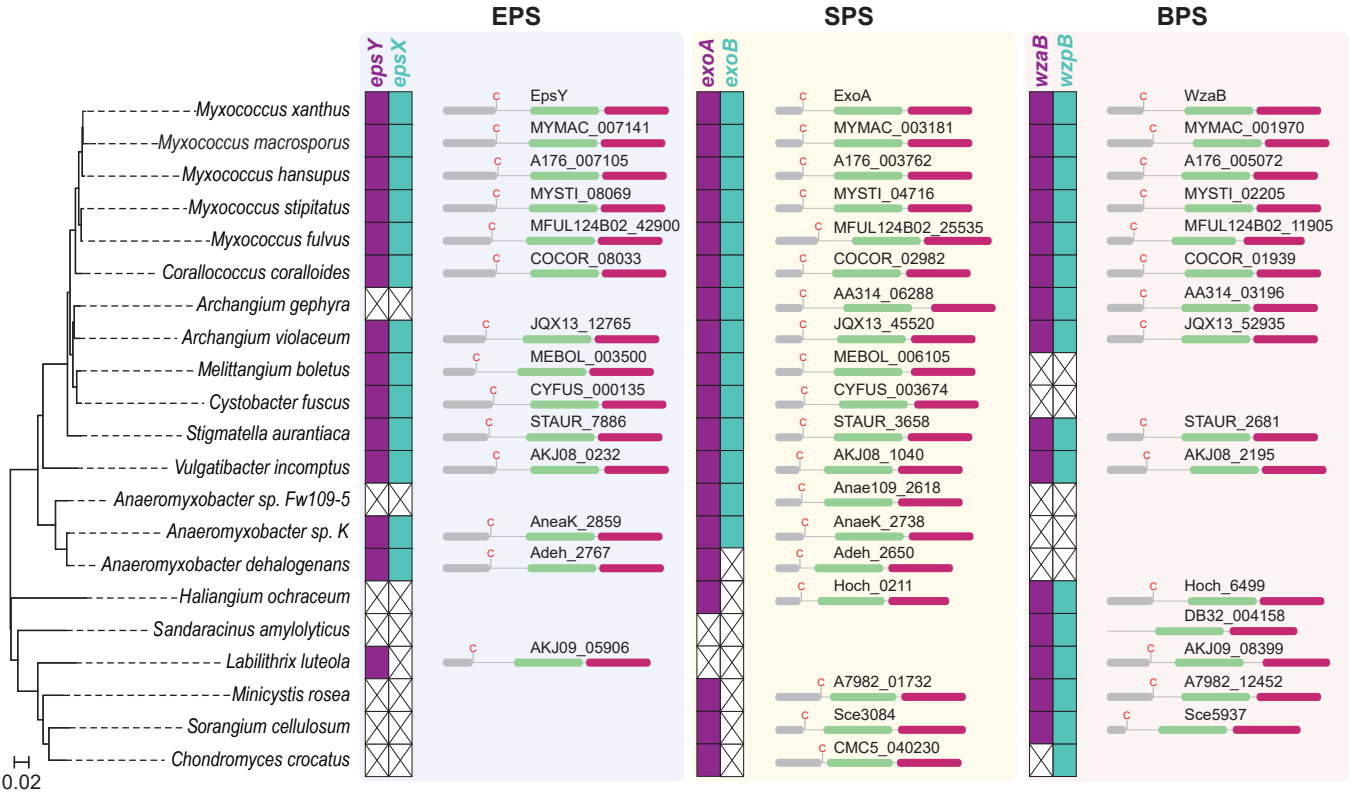

Supplement: FIG S6 [file mbio.02032-22-s0006.pdf]

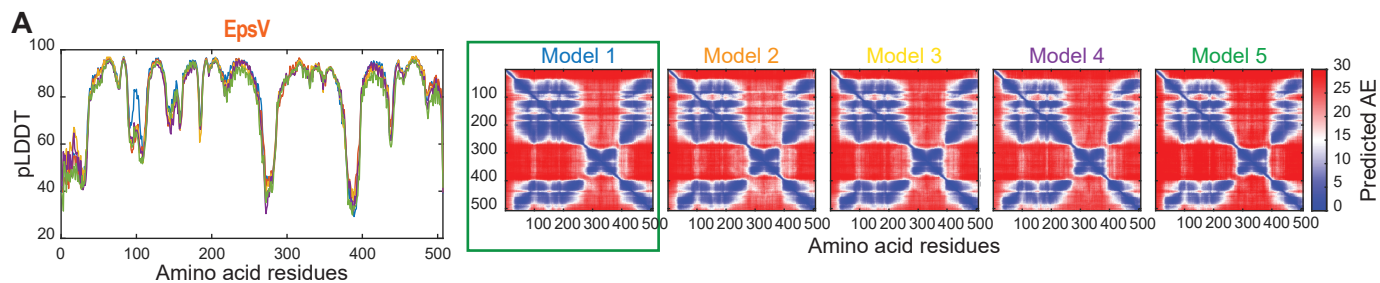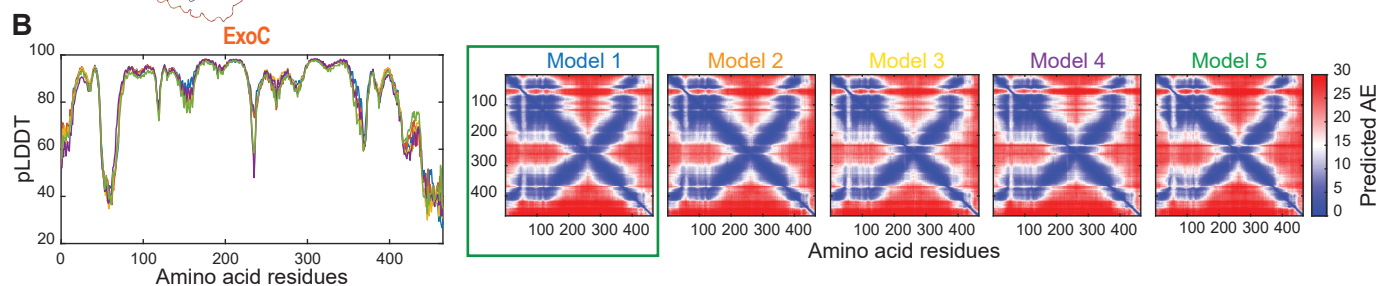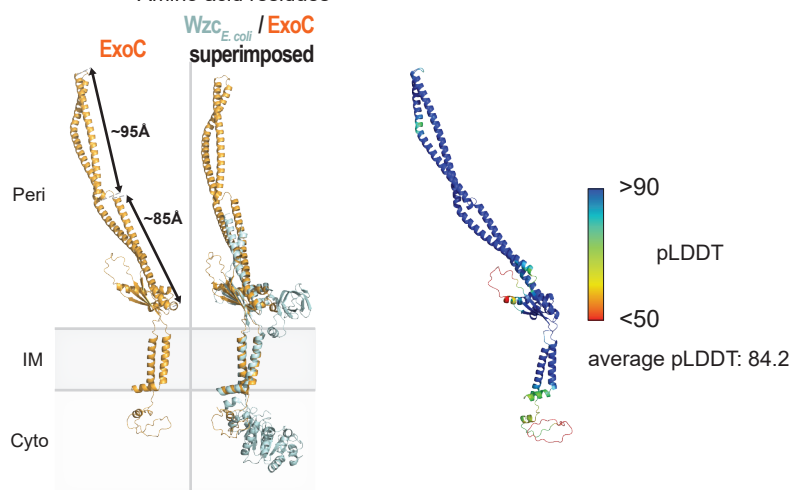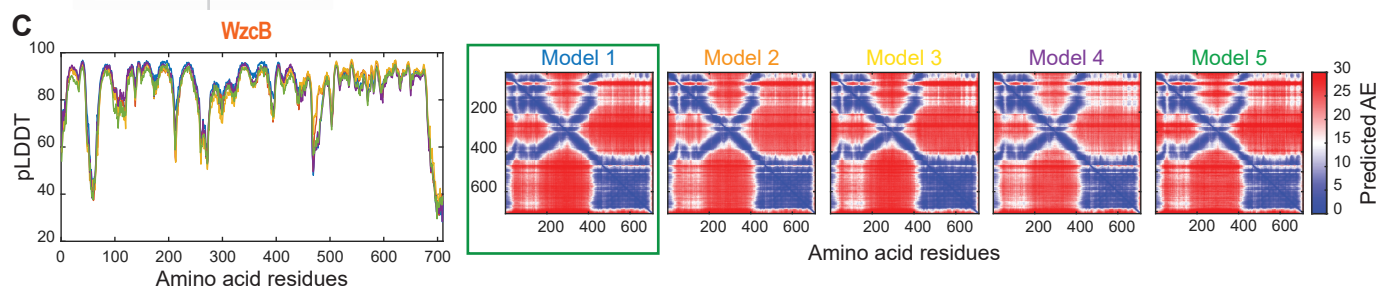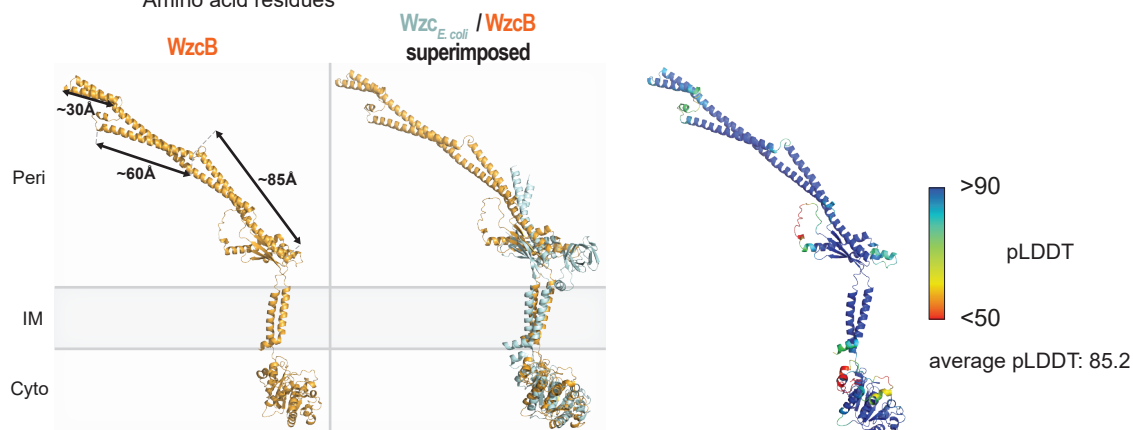

Supplement: FIG S7 [file mbio.02032-22-s0007.pdf]

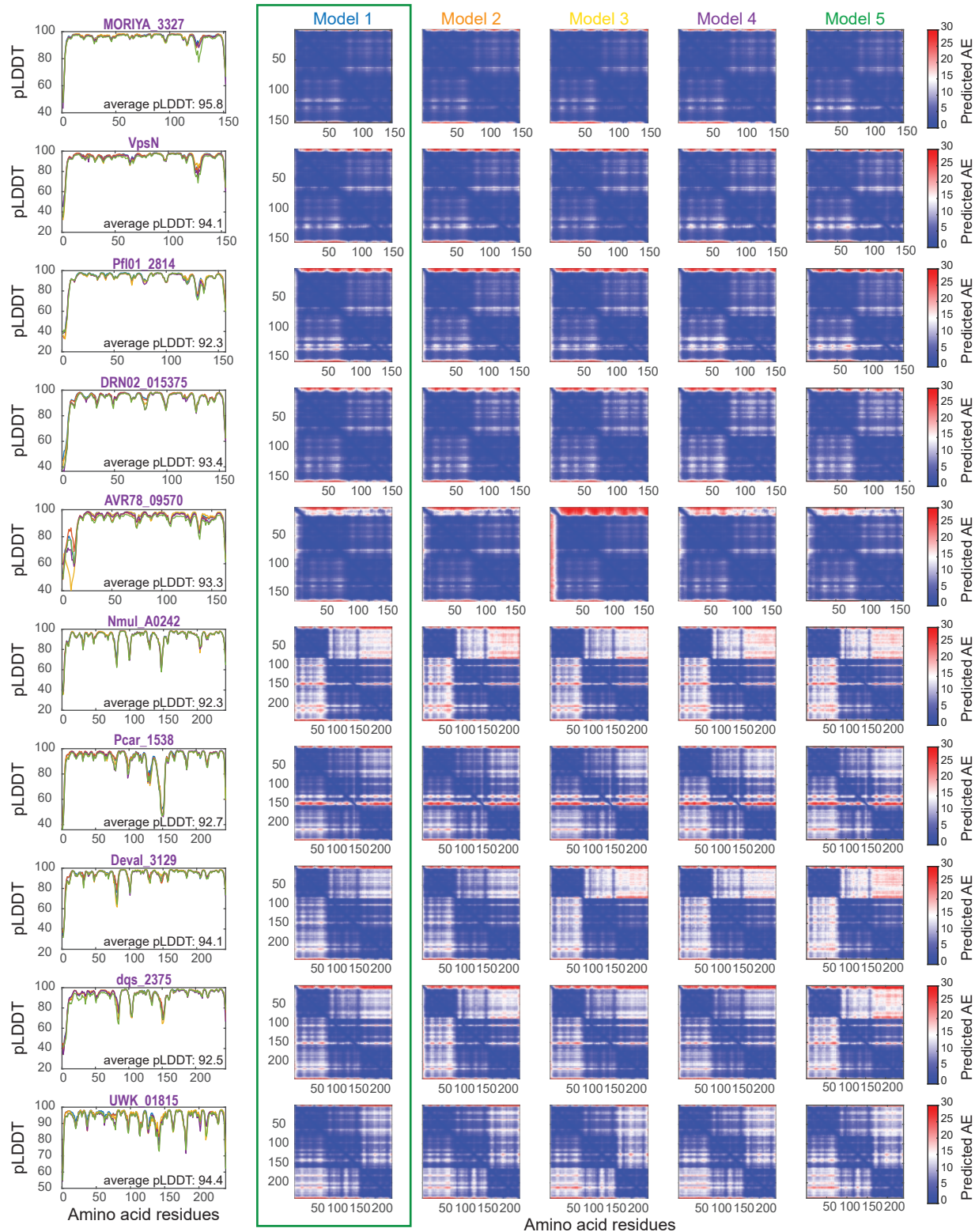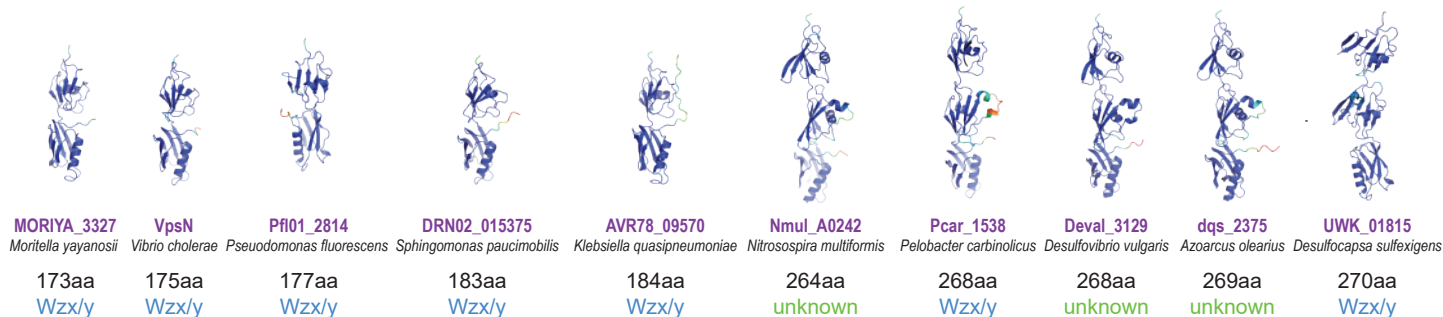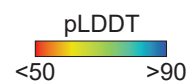

Supplement: FIG S8 [file mbio.02032-22-s0008.pdf]
