# Supplementary material for: Evidence for a Widespread Third System for Bacterial Polysaccharide Export across the Outer Membrane Comprising a Composite OPX/β-Barrel Translocon
Source: mBio. 2022 Aug 16;13(5):e02032-22. doi: 10.1128/mbio.02032-22 (PMC9601211; doi:10.1128/mbio.02032-22)
Supplement: FIG S3 [file mbio.02032-22-s0003.pdf]

A

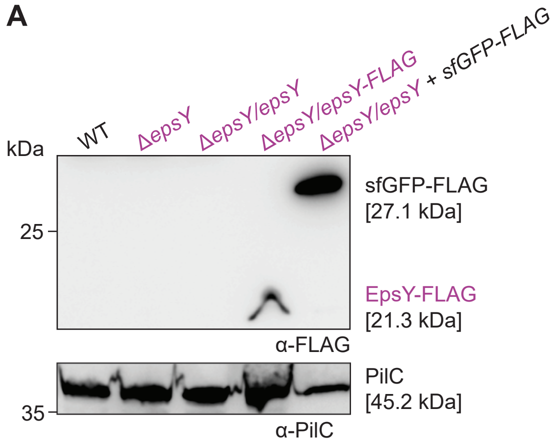

B

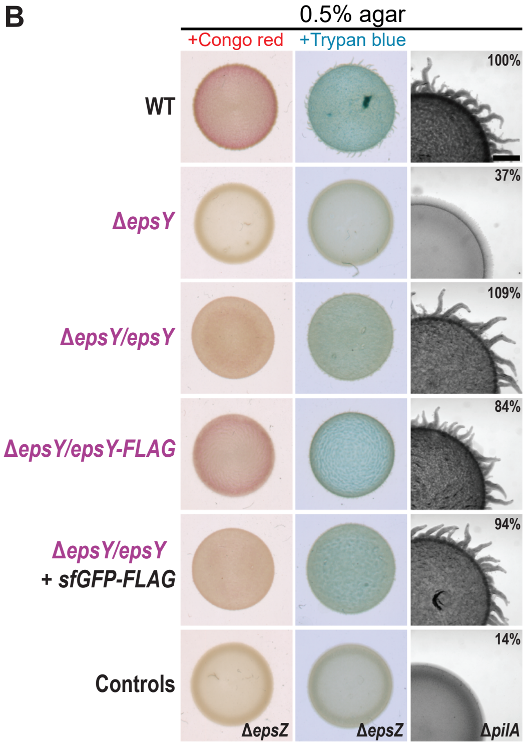

C

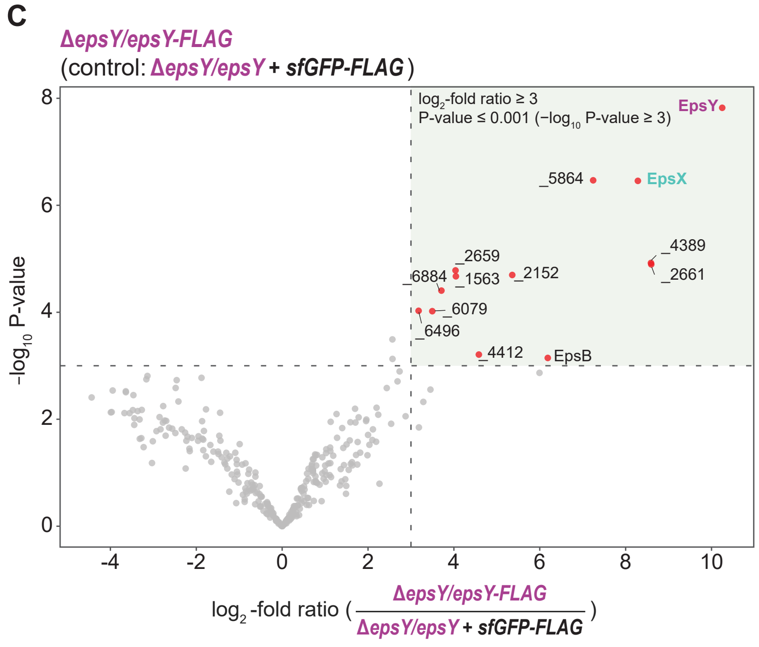

D

Protein name	Function	Signal peptide
EpsY	OPX protein	SPII
MXAN_2661	5´ nucleotidase	SPI
MXAN_4389	Catalase	SPI
EpsX	18-stranded OM β-barrel protein	SPI
MXAN_5864	Hypothetical protein	TAT
EpsB	Glycoside hydrolase	No
MXAN_2152	Oxidoreductase	No
MXAN_4412	Hypothetical protein	No
MXAN_1563	Alkyl hydroperoxide reductase	No
MXAN_2659	Hypothetical protein	SPI
MXAN_6884	Hypothetical protein	No
MXAN_6079	Oxidoreductase	No
MXAN_6496	Thiol peroxidase	No
