# Supplementary material for: Evidence for a Widespread Third System for Bacterial Polysaccharide Export across the Outer Membrane Comprising a Composite OPX/β-Barrel Translocon
Source: mBio. 2022 Aug 16;13(5):e02032-22. doi: 10.1128/mbio.02032-22 (PMC9601211; doi:10.1128/mbio.02032-22)
Supplement: TABLE S1 [file mbio.02032-22-s0009.docx]

**Table S1.** Oligonucleotides used in this work^1^

| **Primer name** | **Sequence 5‘-3‘** | **Brief description** |
| --- | --- | --- |
| 7418-A | TTTGGTACCGGGTGCGCATCACCGTGG | For Δ*epsX* |
| 7418-B | CCGCACGGACGCGACGGTGAGGACCGT | For Δ*epsX* |
| 7418-C | ACCGTCGCGTCCGTGCGGCAAACTGGT | For Δ*epsX* |
| 7418-D | TTTTCTAGAAGGAACCAGTGCCGCAGC | For Δ*epsX* |
| 7418-E | ATGCTTTCGGCGCTGGGC | For Δ*epsX* |
| 7418-F | GTTGCGCTGCGTCAGCAT | For Δ*epsX* |
| 7418-G | ACACCGACGTCACCCCGC | For Δ*epsX* |
| 7418-H | GGATGCTGTCCCCACGAC | For Δ*epsX* |
| 7418-PpilA-for | AAATCTAGAGTGCTGGGGACGGTCCTCA | For complementation of Δ*epsX* |
| 7418-Pnat/PpilA*-*rev | CCCAAGCTTTCAGAGAATACCAGTTTGCCG | For complementation of Δ*epsX* |
| epsY-PpilA-for | ATCGTCTAGAGTGAGGAGAGTTCCACCGCT | For EpsY-FLAG |
| epsY-PpilA-rev | ATCGAAGCTTTTATTCCACCACCACCACGT | For EpsY-FLAG |
| epsY-FLAG43-for | GACTACAAGGACGACGACGACAAGTACACCTGGGTGGAT | For EpsY-FLAG |
| epsY-FLAG43-rev | CTTGTCGTCGTCGTCCTTGTAGTCCTTGCCCAGGCCCCG | For EpsY-FLAG |
| 7417-q-for-2 | AGGACTACATCAACCACCCC | RT-qPCR for *epsY* |
| 7417-q-rev-2 | TGACGAAGATGCGGTCCTTG | RT-qPCR for *epsY* |
| 7418-q-for-5 | CTCCTGGGCCTGGAAATTCG | RT-qPCR for *epsX* |
| 7418-q-rev-5 | CATGTGCTGGATTTCGGTGC | RT-qPCR for *epsX* |
| 7421-q-for-2 | CGACGCGGTCTTCTTTTTGA | RT-qPCR for *epsV* |
| 7421-q-rev-2 | CATGATTTTGCTGACGCCCA | RT-qPCR for *epsV* |

^1^ Underlined sequences indicate restriction sites.
