# Supplementary material for: Evidence for a Widespread Third System for Bacterial Polysaccharide Export across the Outer Membrane Comprising a Composite OPX/β-Barrel Translocon
Source: mBio. 2022 Aug 16;13(5):e02032-22. doi: 10.1128/mbio.02032-22 (PMC9601211; doi:10.1128/mbio.02032-22)
Supplement: TABLE S2 [file mbio.02032-22-s0010.docx]

**Table S2.** Fully sequenced myxobacterial genomes used for the 16S RNA tree

| **Species and strain name** |
| --- |
| *Anaeromyxobacter dehalogenans* 2CP-C |
| *Anaeromyxobacter* sp. Fw109-5 |
| *Anaeromyxobacter* sp. K |
| *Archangium gephyra* DSM 2261 |
| *Archangium violaceum* Cb SDU34 |
| *Chondromyces crocatus* Cm c5 |
| *Corallococcus coralloides* DSM 2259 |
| *Cystobacter fuscus* DSM 52655 |
| *Haliangium ochraceum* DSM 14365 |
| *Labilithrix luteola* DSM 27648 |
| *Melittangium boletus* DSM 14713SG |
| *Minicystis rosea* DSM 24000 |
| *Myxococcus macrosporus* DSM 14675 |
| *Myxococcus hansupus* (*Myxococcus* sp. mixupus) |
| *Myxococcus stipitatus* DSM 14675 |
| *Myxococcus xanthus* DK1622 |
| *Sandaracinus amylolyticus* DSM 53668 |
| *Sorangium cellulosum* So ce 56 |
| *Stigmatella aurantiaca* DW4/3-1 |
| *Vulgatibacter incomptus* DSM 27710 |
